# Neuronal guidance genes in health and diseases

**DOI:** 10.1093/procel/pwac030

**Published:** 2022-07-15

**Authors:** Junichi Yuasa-Kawada, Mariko Kinoshita-Kawada, Yoshio Tsuboi, Jane Y Wu

**Affiliations:** Department of Neurology, Fukuoka University, 7-45-1 Nanakuma, Johnan-ku, Fukuoka 814-0180, Japan; Department of Neurology, Fukuoka University, 7-45-1 Nanakuma, Johnan-ku, Fukuoka 814-0180, Japan; Department of Neurology, Fukuoka University, 7-45-1 Nanakuma, Johnan-ku, Fukuoka 814-0180, Japan; Department of Neurology, Center for Genetic Medicine, Lurie Cancer Center, Northwestern University Feinberg School of Medicine, Ward Building Box 6117, 303 E. Chicago Avenue, Chicago, Illinois 60611, USA

**Keywords:** axon guidance, neuronal migration, synaptogenesis, neural circuit formation, neural mapping, cell-cell communications, angiogenesis, organogenesis, cancer metastasis

## Abstract

Neurons migrate from their birthplaces to the destinations, and extending axons navigate to their synaptic targets by sensing various extracellular cues in spatiotemporally controlled manners. These evolutionally conserved guidance cues and their receptors regulate multiple aspects of neural development to establish the highly complex nervous system by mediating both short- and long-range cell–cell communications. Neuronal guidance genes (encoding cues, receptors, or downstream signal transducers) are critical not only for development of the nervous system but also for synaptic maintenance, remodeling, and function in the adult brain. One emerging theme is the combinatorial and complementary functions of relatively limited classes of neuronal guidance genes in multiple processes, including neuronal migration, axonal guidance, synaptogenesis, and circuit formation. Importantly, neuronal guidance genes also regulate cell migration and cell–cell communications outside the nervous system. We are just beginning to understand how cells integrate multiple guidance and adhesion signaling inputs to determine overall cellular/subcellular behavior and how aberrant guidance signaling in various cell types contributes to diverse human diseases, ranging from developmental, neuropsychiatric, and neurodegenerative disorders to cancer metastasis. We review classic studies and recent advances in understanding signaling mechanisms of the guidance genes as well as their roles in human diseases. Furthermore, we discuss the remaining challenges and therapeutic potentials of modulating neuronal guidance pathways in neural repair.

## Introduction

Precise neuronal positioning and neural wiring is a prerequisite for the functional architecture of the nervous system and its information processing. How the complex neural architectures are formed is a fundamental question in biology.

**Figure 1.**
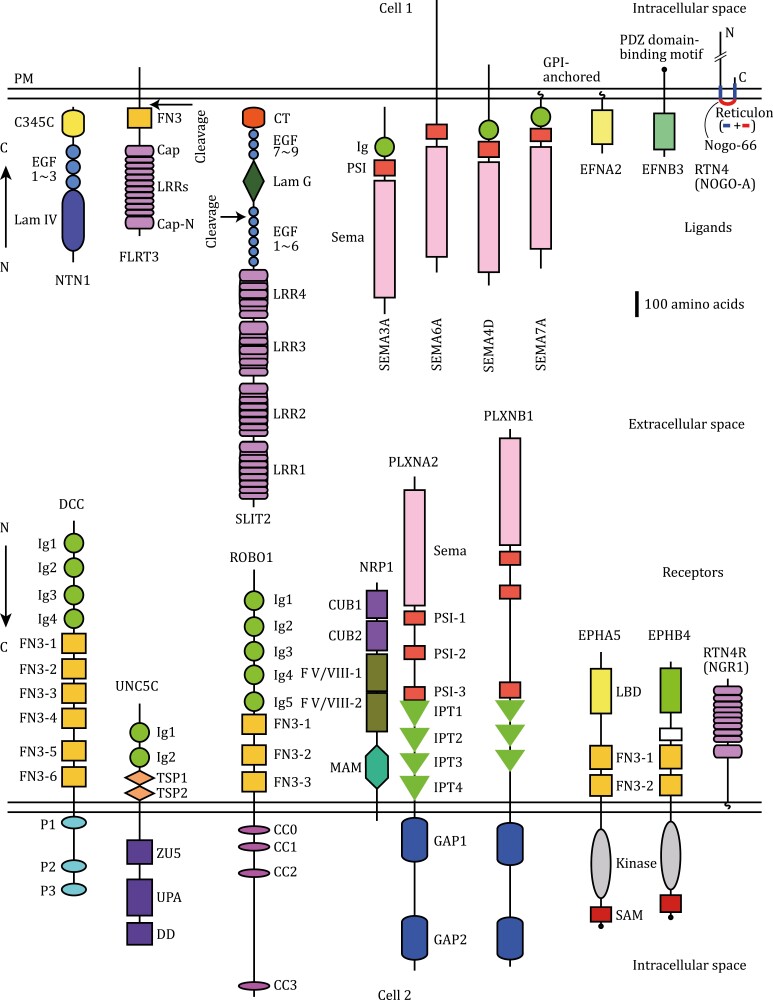
Neuronal guidance cues and receptors. A schematic illustration of guidance cues and receptors based on domain structures annotated by EMBL-SMART. CC: conserved cytoplasmic; CT: C-terminal cysteine knot; CUB: found in C1r, C1s, uEGF, and BMP; DD: death domain; FN3: fibronectin type 3; F V/VIII: factors V and VIII (coagulation factors V and VIII); GAP: GTPase-activating protein; Ig: immunoglobulin-like; IPT: immunoglobulin-plexin-transcription; Lam: laminin-type; LBD: ligand-binding domain; LRR: leucine-rich repeat; MAM: present in meprin, A5, receptor protein tyrosine phosphatase mu; PSI: plexin-semaphorin-integrin; SAM: sterile alpha motif; TSP: thrombospondin type 1; UPA: UNC5-PIDD-ankirin; ZU5: present in ZO-1 and UNC5. The LRR domain structure of FLRT3 is modified based on Seiradake et al. (2014). Each LRR domain of SLIT2 is composed of multiple LRR assemblies. EFNBs and EPH receptors C-terminally bear PDZ domain (the scaffold domain shared by Postsynaptic density-95)-binding motifs. Reticulon-4 (RTN4)/NOGO interacts with RTN4 receptors (RTN4Rs). Note that not included in the diagram are non-canonical guidance cues and the recent finding of interaction between RTN4R and brain-specific angiogenesis inhibitors (BAIs) (Wang et al., 2021).

**Figure 2.**
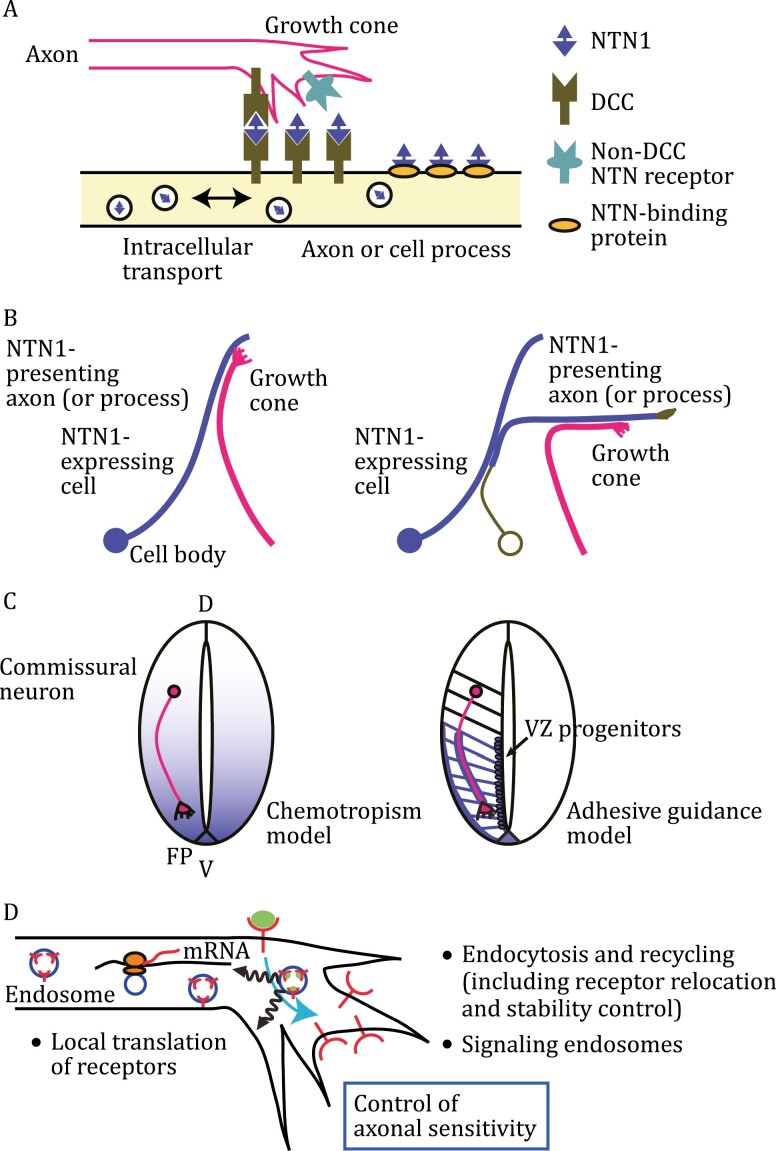
Mechanisms regulating presentation of neuronal guidance cues and expression/function of their receptors. (A) Axons sense guidance cues (e.g., NTN1), which are presented on axonal membranes. NTN1, DCC, non-DCC NTN receptors and NTN-binding proteins are depicted on the right. NTN1 has multiple receptor-binding sites (Meijers et al., 2020). (B) A NTN1 (blue)-expressing axon presents NTN1 to a growing axon (pink) (left panel). Alternatively, a non-NTN1-expressing cell captures NTN1, presents NTN1 to a growing axon and direct its growth (right panel). (C) A chemotropism-based model (left) versus an adhesive guidance model (right) for axon guidance at the midline. In the right panel, radial glial progenitors at the ventricular zone (VZ) present NTN1 to growing commissural axons (pink), using DCC distributed on their long radial processes (blue lines). For clarity, radial glia and their processes are illustrated on the left half of the spinal cord. D: dorsal; FP: floorplate; V: ventral. (D) Local-translation and endocytic recycling-based control of axonal sensitivity to guidance cues. Internalized, cue-bound receptors can continue to emanate signals from signaling endosomes, which may be different from those from the plasma membrane-localized receptors. In addition, the efficiency of intracellular signaling cascades is also modulatable, affecting the overall cellular responses.

During neural development, the growth cone, a sensory apparatus at the axon tip, is responsible for axon navigation to its target (Lowery and Van Vactor, 2009). Migrating neurons also have growth cone-like structures at the tip of their leading processes. Growth cones sense secreted and membrane-bound guidance cues, which can be instructive or permissive, and attractive or repulsive, dependent on the context (Figs. 1, 2A and 2B) (Tessier-Lavigne and Goodman, 1996; Guan and Rao, 2003; Dickson and Zou, 2010; Chédotal, 2019; Dorskind and Kolodkin, 2021; Zang et al., 2021). Neuronal pathfinding decisions rely on signal transduction from extracellular cues, via their receptors, to intracellular molecular cascades that control cytoskeletal reorganization, membrane remodeling, and vesicular trafficking (Vitriol and Zheng, 2012).

Canonical guidance cues, including netrins, Slits, semaphorins, and ephrins, together with their receptors, play critical roles in not only axon guidance and neuronal migration (Wu et al., 1999) but also dendrite formation (Polleux et al., 2000; Dong et al., 2015), neuronal polarization (Shelly et al., 2011), synaptic target choice, synaptogenesis, and plasticity (Sanes and Zipursky, 2020; Glasgow et al., 2021; Südhof, 2021). Furthermore, neuronal guidance genes regulate numerous processes outside the nervous system, such as organogenesis and tissue homeostasis. Dysfunction of guidance genes has been associated with various diseases, including developmental/neuropsychiatric disorders, cardiovascular diseases, inflammatory diseases, and cancer. This review summarizes our current understanding of neuronal guidance signaling, its functions during neural development and organogenesis/homeostasis and its roles in disease pathogenesis. Finally, we discuss therapeutic potentials of modulating neuronal guidance pathways and future challenges toward a deeper understanding of neuronal guidance in health and diseases.

## Historical overview and basic concepts

In the 19th century, Ramón y Cajal postulated that axons might be guided by diffusible cues (Cajal, 1995). Sperry (1963) proposed the chemoaffnity hypothesis that position-specific chemical labels mediate axon targeting to establish neural maps. Since the 1980s, many experimental models were developed to analyze axonal behavior. First, collapsins/semaphorins, chemorepellents for axons, were identified by using a “growth cone collapse assay”. cDNA cloning of collapsin/semaphorin3A (SEMA3A) in chickens (Luo et al., 1993) and several semaphorins in *Drosophila* and humans (Kolodkin et al., 1993) sparked the molecular studies of axon guidance genes (Fig. 1). The evidence that chemotropic cues released from ventral midline floorplate cells attract commissural axons (Fig. 2C, left) led to the discovery of netrins as chemoattractants (Tessier-Lavigne et al., 1988; Serafini et al., 1994; Kennedy et al., 1994).

To study topographic axon projections from the retina to the optic tectum in chickens (retinotectal projection), a “stripe assay” was devised to culture retinal explants on alternating cell-membrane carpets prepared from rostral (anterior) and caudal (posterior) tectum (Walter et al., 1987). The observation that temporal, but not nasal, axons were repelled by caudal tectal cell-membranes led to the later identification of membrane-anchored, topographic repellents, ephrins (Kania and Klein, 2016).

A genetic screen for axon pathway formation identified two *Drosophila* lines defective in midline axon guidance: one showing multiple-time midline re-crossing of commissural axons (axonal populations that connect the left and right sides of the nervous system), named *roundabout* (*robo*), and the other lacking the commissure, named *commissureless* (*comm*) (Seeger et al., 1993). Subsequently, the *Robo* genes were cloned (Fig. 1) (Kidd et al., 1998; Zallen et al., 1998). SLITs were then identified as ligands for ROBO to repel axons at the midline (Brose et al., 1999; Kidd et al., 1999; Li et al., 1999; Zou et al., 2000). Comm suppresses axonal ROBO levels and SLIT sensitivity in *Drosophila* (Keleman et al., 2005). Remarkably, SLIT also repels neurons that migrate from the anterior subventricular zone (SVZa) in the telencephalon to the olfactory bulb (Wu et al., 1999). Thus, the concept that axon guidance cues can direct migration of neuronal cell bodies was established.

Although responses to cues are mediated by guidance receptor activation, manipulating the balance of intracellular signaling molecules can switch cellular responses. For example, activating the cGMP pathway over the cAMP pathway, the axonal response to netrin-1 switches from attraction to repulsion, whereas the response to SEMA3A is converted from repulsion to attraction (Ming et al., 1997; Song et al., 1998). Ca^2+^ signaling also modulates axonal responses (Gomez and Zheng, 2006). The balance between cAMP and cGMP, together with Ca^2+^ mobilization through various Ca^2+^ channels, determines attractive or repulsive responses of axons to guidance cues. Therefore, activation of the same receptor can trigger either attraction or repulsion, depending on the responding neuron’s context. Such mechanisms can operate simultaneously in a single neuron. In cortical/hippocampal neurons, SEMA3A acts as a repellent for axons but an attractant for apical dendrites and regulates neuronal polarity by controlling the cAMP/GMP balance (Polleux et al., 2000; Shelly et al., 2011).

Guidance cues also regulate the morphogenesis of axons or dendrites. For example, SLIT regulates axon/dendritic branching (secondary sprouting along the neurite shaft or at the terminal) (Wang et al., 1999; Whitford et al., 2002). Thus, guidance mechanisms regulate both neuronal pathfinding and axon/dendritic morphogenesis.

The report that SLIT inhibits chemokine SDF-1 (CXCL12)-induced leukocyte chemotaxis (Wu et al., 2001) opened the avenue to address roles of neuronal guidance genes in regulating migration of non-neuronal cells. Furthermore, a chemotactic mechanism drives breast cancer metastasis toward CXCL12-releasing target organs, such as lymph nodes (Müller et al., 2001). These studies inspired examination of cross-communications between signaling pathways mediated by guidance cues and by chemokines/growth factors in modulating cancer cell behavior. Subsequent studies revealed that neuronal guidance genes may negatively or positively contribute to tumorigenesis and cancer metastasis (Mehlen et al., 2011; Worzfeld and Offermanns, 2014; Kania and Klein, 2016). This fueled further research to examine how neuronal guidance genes function outside the nervous system to regulate organogenesis and tissue homeostasis.

Below, we discuss several general mechanisms of neuronal guidance. Many guidance cue-producing cells present these cues locally. However, guidance cues, such as netrin-1, can be delivered along axons or processes of neural progenitors and presented in a long distance by guidance receptors or cue-binding proteins. Such molecules capture secreted cues to form neurite-growth substrates (Fig. 2A–C) (Dominici et al., 2017). The cue-presentation system was originally discovered in *Drosophila*, in which Frazzled, a homologue of mammalian netrin receptor, Deleted in Colorectal Cancer (DCC), captured netrin secreted by other cells and then presented it to target cells (Hiramoto et al., 2000). Thus, even cells not producing guidance cues are able to present them to direct neuronal pathfinding in both invertebrates and vertebrates (Fig. 2B).

Different neurons express distinct repertoires of guidance receptors and intracellular signaling components. Expression of neuronal guidance genes is under spatiotemporal control at the transcriptional, post-transcriptional, translational, and post-translational levels. mRNA translation occurs not only in cell bodies but also in neurites (Fig. 2D) (Fernandopulle et al., 2021). Local mRNA translation efficiency is modulated, allowing timely production of “new” guidance receptors when axons reach guideposts (e.g. the ventral midline). In addition, intracellular trafficking of “old” receptors regulates their axon-surface levels and axonal responses to guidance cues. While endocytosis of receptors may lead to their degradation, causing desensitization or response termination, endocytosis can also initiate, maintain, and even potentiate receptor signaling (Sigismund et al., 2021). Endocytosis-triggered intracellular trafficking and signaling are typical signal-transduction modes in neuronal guidance, similar to many other biological processes (Fig. 2D). For example, upon midline crossing, commissural axons are sensitized to SLIT itself by driving ROBO1 endocytosis and recycling back to the cell membrane (Kinoshita-Kawada et al., 2019). Therefore, axons change their responsiveness to guidance cues through local translation and receptor trafficking, as well as tuning of intracellular cascades.

**Figure 3.**
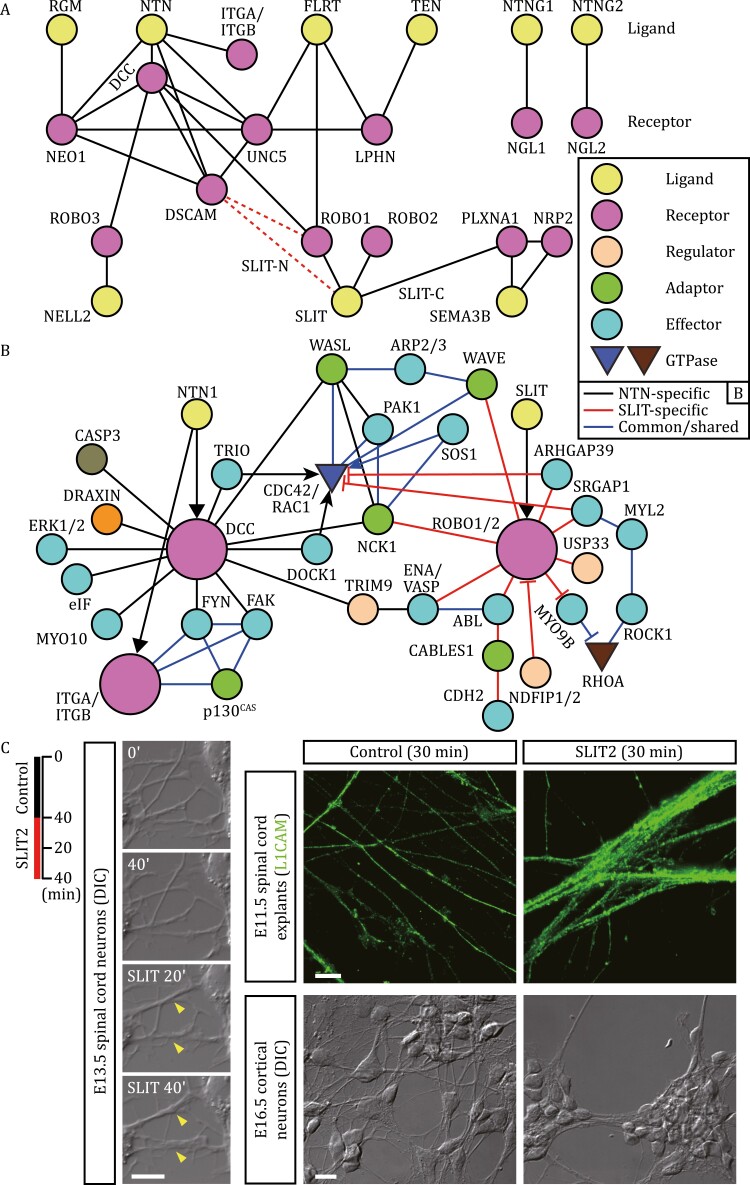
Networks of netrin and Slit signaling pathways. (A) Ligand-receptor relationships of NTN, FLRTs and SLIT pathways. Also depicted are the interactions of PLXNA1 with SLIT-C, SEMA3B and NRP2. The interactions of DSCAM with SLIT-N or with ROBO1 in vertebrates remain to be verified (shown by red dotted lines). (B) The intracellular signaling cascades triggered by NTN1-DCC and SLIT-ROBO1 pathways that share several key signaling components. The signaling networks are presented based on biochemical interactions. (C) Axon fasciculation upon exposure to SLIT2 (25 pmol/L) in primary cultures of mouse dorsal spinal cord neurons (time-lapse differential interference contrast [DIC] imaging in live cells) and cortical neurons (DIC images of fixed cells) and in floorplate-containing spinal cord explant culture (immunostained with anti-L1CAM, a post-crossing commissural axon marker) (see Kinoshita-Kawada et al., 2019). In these cultures, axon fasciculation rapidly proceeds upon SLIT2 stimulation. In time-lapse imaging (left panels), the same neurons were stimulated sequentially with a control, then with SLIT2 (two axon bundles formed are marked with arrowheads). Note that dissociated cortical neurons also exhibited cell-adhesive responses (right lower panels). Scale bars: 10 μm.

**Figure 4.**
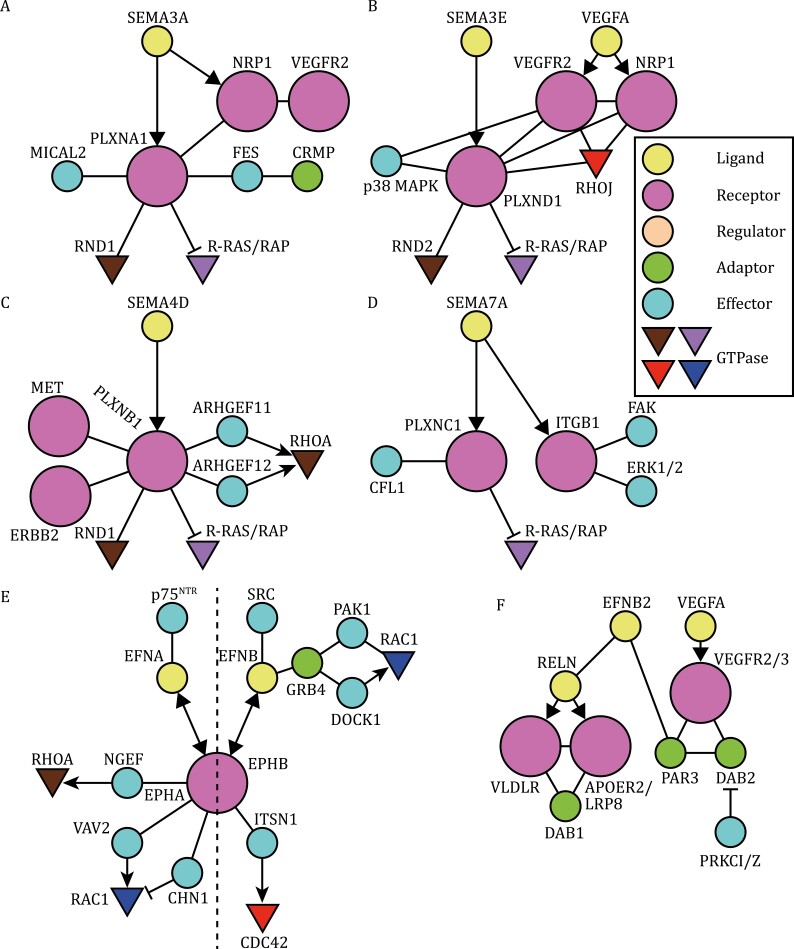
Networks of semaphorin and ephrin signaling pathways. Semaphorin and ephrin pathways are substantially heterogeneous, although they share several common signaling cascades (see text for details). (A–D) Semaphorin pathways. (E–G) Ephrin pathways. Bidirectional and reverse signaling pathways are also depicted.

Cross-communication between different receptors and/or signal transducers also coordinates neuronal responses to multiple cues (See Figs. 3 and 4). These mechanisms enable hierarchical or reciprocal regulations of different guidance pathways (Zang et al., 2021). For example, SLIT-induced DCC-ROBO1 interactions suppress axonal responsiveness to netrin-1 (Stein and Tessier-Lavigne, 2001).

Furthermore, guidance cues/receptors are frequently associated with the adhesion machinery. SLIT and ephrin signaling pathways suppress cadherins; whereas netrins and semaphorins utilize integrins as receptors and modulate integrin function. In many situations, growth cones sense both guidance and adhesive cues, and integrate the overall inputs. Thus, multi-layered mechanisms ensure that individual neurons and axons respond to multiple cues in spatiotemporally organized manners.

## Guidance cues, receptors, and signaling pathways

Guidance cues and receptors have multiple families of binding partners. Here, we comparatively examine several representative guidance cues and downstream signaling pathways, and discuss how they crosstalk to determine cellular behavior. The major outputs of various neuronal guidance signaling pathways converge onto different families of small GTPases. They cycle between an inactive GDP-bound form and an active GTP-bound form (Niftullayev and Lamarche-Vane, 2019; Müller et al., 2020). Small GTPases are activated by guanine nucleotide exchange factors (GEFs) and switched off by GTPase-activating proteins (GAPs). Activated GTPases bind to effectors to regulate diverse cellular processes, including cytoskeletal reorganization, membrane dynamics, and cell–cell/cell–substrate interactions.

### Signaling pathways by NTNs and FLRTs

Netrins (NTNs) are laminin-like domain-containing guidance cues (Serafini et al., 1994; Kennedy et al., 1994). The netrin family consists of secreted netrin-1 to -5, and glycosylphosphatidylinositol (GPI)-anchored netrin-G1 and -G2 (Lai Wing Sun et al., 2011; Meijers et al., 2020). Fibronectin leucine-rich repeat transmembrane proteins (FLRTs) act as cell-adhesion regulators and as guidance cues (Figs. 1 and 3A) (Yamagishi et al., 2011). Secreted NTNs are sensed as attractants or repellents by DCC and neogenin1 (NEO1), or as repellents by uncoordinated-5 (UNC5) receptors (UNC5A–D) (Lai Wing Sun et al., 2011). These receptors belong to the immunoglobulin (Ig) superfamily of cell-adhesion molecules (CAMs). UNC5s act alone, or bind to DCC or NEO1, to form repulsive netrin receptors. In the absence of UNC5, DCC, and NEO1 act as attractive netrin receptors. Structurally, netrin-DCC (or NEO1) complexes form netrin-receptor-netrin-receptor continuous assembly (Xu et al., 2014; Robinson et al., 2021).

UNC5s act as repulsive receptors not only for netrins but also for proteolytically shed or full-length FLRTs (Fig. 3A) (Yamagishi et al., 2011; Seiradake et al., 2014). FLRTs exert FLRT-UNC5-mediated repulsion and FLRT-FLRT-mediated cell-adhesion, enabling combinatorial control of clustering and segregation of migrating cells. Thus, netrins and FLRTs differentially induce attractive, repulsive, or adhesive responses of various cell types.

Netrins and FLRTs also regulate synaptic specificity and synaptogenesis (Poon et al., 2008; reviewed by Sanes and Zipursky, 2020; Glasgow et al., 2021; Südhof, 2021). Full-length FLRTs act as ligands for Latrophilins (LPHN1–3), adhesion G protein-coupled receptors (adhesion-GPCRs) that organize excitatory synapse formation (Sando et al., 2019). Teneurins (TENs) are homophilic CAMs (Berns et al., 2018) and also ligands for LPHNs (Sando et al., 2019; del Toro et al., 2020). Transsynaptic, coincident binding of presynaptic FLRTs and TENs to postsynaptic LPHNs ensure the specificity of excitatory synapses (Fig. 3A) (Sando et al., 2019). Interestingly, this supercomplex also mediates repulsive signals for migrating neurons (del Toro et al., 2020). It is unclear how TEN-FLRT-LPHN-mediated adhesion and repulsion are balanced for synapse formation and neuronal migration.

NEO1 binds to netrins and repulsive guidance molecules (RGMs) (Fig. 3A) (Monnier et al., 2002). NEO1 mediates attraction upon netrin-1 binding, but repulsion upon RGM binding. Simultaneous binding of netrin-1 and RGM to NEO1 silences their outputs (Robinson et al., 2021). This is a unique guidance signal-cancelling mechanism that may prevent disadvantageous competition between attraction and repulsion. Down Syndrome CAM (DSCAM) also acts as a receptor for netrin and for SLIT to regulate axon pathfinding/branching and neuronal migration (Ly et al., 2008; Dascenco et al., 2015; Brignani et al., 2020). In addition, netrins bind to integrins and regulate their activity. Conversely, integrins induce the attraction-to-repulsion transition of netrin responses (Höpker et al., 1999; Yebra et al., 2003).

GPI-anchored netrins, netrin-G1 and -G2, bind to their receptors, netrin-G ligand-1 (NGL-1 [LRRC4C]), and NGL-2 (LRRC4), respectively (Woo et al., 2009). Netrin-G-NGL signaling promotes presynaptic differentiation and excitatory synaptogenesis. Combinations of cell-recognition modules, including TEN-FLRT-LPHN and netrin-G-NGL pathways, may confer synaptic specificity (Sanes and Zipursky, 2020).

Netrins regulate several intracellular signaling pathways. The DCC intracellular domain (ICD) contains evolutionally conserved, low-complexity regions (LCRs) (P1–3 motifs; Fig. 1). The P1–3 provide multivalent scaffolds for interacting with various signal mediators. In the intial signaling phase, DCC interacts with focal adhesion kinase (FAK) and SRC family tyrosine kinases, including FYN (Fig. 3B) (Liu et al., 2004; Xu et al., 2018). Furthermore, p130^CAS^ and a DCC-associated RAC1-GEF, DOCK1 (DOCK180) mediates netrin attraction (Liu et al., 2007; Li et al., 2008). Interestingly, they are also integrin signaling components. Consistently, netrin immobilization onto the substrate and FAK- and actomyosin-dependent mechanotransducion are required for netrin attraction (Moore et al., 2012). Therefore, netrin signaling simultaneously regulates neuronal pathfinding and integrin-mediated adhesion. It is still under debate whether netrins exert attractive responses in a diffusible or immobilized state (see below).

Upon netrin-DCC binding, the SH3/SH2 adaptor NCK1 associates with proline-rich motifs of the DCC-ICD (Lai Wing Sun et al., 2011). NCK1 provides a scaffold to further assemble netrin signal mediators and effectors, including WASL (N-WASP). WASL activates the ARP2/3 complex to stimulate actin filament nucleation and neurite outgrowth. PAK1, a serine/threonine kinase and RAC1-GTP effector, is also recruited to the DCC-NCK1 complex (Lai Wing Sun et al., 2011). Multivalent, LCR-containing DCC (P1–3), NCK1, and WASL may control liquid–liquid phase separation of netrin signaling components. Furthermore, DCC-associated FAK, SRC kinases, and PAK1 may promote phase separation through phosphorylation. Collectively, upon netrin-DCC binding, both the extracellular netrin-DCC polymer-like assembly and the intracellular signaling network generate a sharp transition from the inactive to the active state.

Enabled/vasodilator-stimulated phosphoprotein (ENA/VASP) family members promote actin polymerization. TRIM9, a DCC-associated E3 ubiquitin ligase, and ENA/VASP are localized to filopodial tips of growth cones (Menon et al., 2015). TRIM9 ubiquitinates VASP, reducing VASP localization at filopodial tips. Upon netrin stimulation, VASP is deubiquitinated and accumulates at filopodial tips, selectively stabilizing filopodia through actin polymerization, which may cause axon turning.

Roles of DCC and UNC5 in neuronal death remains controversial (Williams et al., 2006). Although netrin-DCC signaling activates p38 mitogen-activated protein kinase (MAPK), which regulates apoptosis by activating CASP3, an apoptotic executioner caspase (Campbell and Holt, 2003), it is still unclear how apoptotic netrin signaling regulates axon pathfinding (Kellermeyer et al., 2018).

### The SLIT signaling pathways

The *Slit* family consists of three *SLITs* in vertebrates and one *Slit* in *Drosophila* (Blockus and Chédotal, 2016). SLITs contain multiple leucine-rich repeats (LRRs) and epidermal growth factor (EGF) repeats. There are four ROBO receptors in vertebrates and three Robos in *Drosophila*. ROBOs belong to the IgCAM superfamily, similar to DCC (Fig. 1). In vertebrates, ROBO3 inhibits ROBO1/2; whereas in *Drosophila*, Robo2 inhibits Robo1 (Blockus and Chédotal, 2016). In most situations, full-length SLIT1–3 act as secreted repellents for ROBO1/2-expressing axons or cells.

Many details concerning SLIT-ROBO pathways came from studying commissural axon projections in developing spinal cords. Commissural neurons extend their axons to cross the midline and project contralaterally. SLITs are expressed in floorplate cells at the midline. The crossing of commissural axons requires precise regulation of ROBO receptors. Ipsilaterally projecting axons maintain ROBO expression at high levels and respond to SLIT, so as not to cross the midline. In contrast, pre-crossing commissural axons express ROBO at low levels and do not respond to SLIT; however, upon reaching and crossing the ventral midline, commissural axons express ROBO at higher levels and are expelled away from the midline by repulsive activities of SLIT, together with SEMA3B/3F (Kidd et al., 1998, 1999; Zou et al., 2000; Keleman et al., 2005; reviewed by Chédotal, 2019). Comm in *Drosophila* and its mammalian homologues, PRRG4 and/or NDFIP1/2, reduce axonal ROBO levels during midline crossing (Justice et al., 2017; Gorla et al., 2019).

Our understanding of midline crossing by commissural axons in mammals was largely from analyzing knockout animals. In *Slit1*/*2*/*3* triple-knockout, *Robo1*/*2* single- or double-knockout mice, commissural axons stall within the midline or just after exiting the midline or exhibit post-crossing misrouting (Jaworski et al., 2010). In *Robo3*-deficient mice, spinal commissural axons do not enter the midline. Thus, ROBO1/2 mediate SLIT repulsion of commissural axons, whereas ROBO3 (specifically, ROBO3.1 isoform) suppresses SLIT sensitivity of commissural axons (Sabatier et al., 2004; Chen et al., 2008). Unexpectedly, ROBO3 forms a complex with DCC and potentiates its function as a attractive netrin receptor (Zelina et al., 2014). Mammalian ROBO3 also bind to neural EGF-like-like-2 (NELL2), which is secreted by motor neurons and repels commissural axons, but not to SLIT (Jaworski et al., 2015). Collectively, ROBO1/2 are regulated so that commissural axons sense SLIT in spatiotemporally precise manners, whereas ROBO3 regulates responsiveness of commissural axons to multiple cues.

SLIT undergoes proteolytic processing to generate an N-terminal (SLIT-N) and C-terminal (SLIT-C) fragments (Brose et al., 1999; Ducuing et al., 2020). In most studies, SLIT-N promoted axonal/dendritic growth and branching (Wang et al., 1999; Whitford et al., 2002; Alavi et al., 2016; Kellermeyer et al., 2020). Although it remains unclear how full-length SLIT/SLIT-N regulates neurite repulsion, growth, or branching, co-receptor molecules may modulate ROBO function. For example, in *Drosophila*, SLIT-N binds to Robo/DSCAM complex to trigger axon branching (Alavi et al., 2016). In contrast, SLIT-C is a repulsive ligand for a semaphorin receptor, PLXNA1 (Delloye-Bourgeois et al., 2015). PLXNA1 senses SLIT-C in the neuropilin (NRP2)-unbound state but senses SEMA3B in the neuropilin-bound state. Thus, upon proteolytic cleavage, SLIT signals are bifurcated to neurite branching/growth-promoting activity of SLIT-N and plexin-mediated repulsive activity of SLIT-C.

The ROBO-ICDs contain evolutionally conserved cytoplasmic (CC) motifs, CC0–3 (Fig. 1). Abelson (ABL), a nonreceptor tyrosine kinase, binds to CC3, phosphorylates CC1 and inhibits Slit-Robo signaling in *Drosophila* (Bashaw et al., 2000). CC2 is a proline-rich conserved binding site for ENA, an ABL substrate (Bashaw et al., 2000), while CC3, another proline-rich stretch, is recognized by SLIT-ROBO GAPs (SRGAP1–3). SRGAPs contain SH3, RhoGAP, and F-BAR domains (Wong et al., 2001; Guerrier et al., 2009). Although SRGAP1 was characterized as a CDC42-GAP in repulsive SLIT-ROBO signaling (Wong et al., 2001), subsequent studies suggested that SRGAPs generally act as RAC1-GAPs (Guerrier et al., 2009). Systems-level data from HEK293T cells revealed that SRGAP1/3 act as RAC1-GAPs, whereas SRGAP2 is directed to RAC1/CDC42 (Müller et al., 2020). SRGAPs play broader biological roles than initially anticipated: neuronal migration, neurite branching, synaptogenesis, cell adhesion, and non-neuronal cellular morphogenesis (Wong et al., 2001; Guerrier et al., 2009; Beamish et al., 2018). Whether SRGAPs regulate SLIT-independent processes remains unclear.

Other downstream signaling molecules have also been characterized. Upon SLIT stimulation, PAK1 is recruited to ROBO1 and activated by RAC1-GTP and NCK1 (Fig. 3B) (Fan et al., 2003). Multiple RhoGEFs/GAPs participate in the signaling. For example, Son of sevenless (SOS1) acts as a RAC1-GEF upon association with ROBO1 via NCK1 (Yang and Bashaw, 2006). ROBO1 binds to the GAP domain of MYO9B, a RhoGAP domain-containing unconventional myosin. SLIT-ROBO binding suppresses MYO9B RhoGAP activity, elevating RHOA-GTP levels (Kong et al., 2015). RHOA-GTP activates Myosin II via RHOA-associated kinase (ROCK1) (Niftullayev and Lamarche-Vane, 2019). ROBO-interacting RhoGEFs/GAPs may enable combinatorial control of actomyosin and adhesion machineries, through spatial regulation of RhoGTPases. WAVE regulatory complex (WRC), a RAC1-GTP effector, is recruited to SLIT-bound ROBO and activates ARP2/3 complex to promote actin polymerization, collaboratively with ENA/VASP (Chaudhari et al., 2021). This cascade is essential for SLIT repulsion and commissural axon midline crossing. Transient filopodial extensions toward SLIT sources depend on the ROBO-ENA/VASP complex and are required for SLIT repulsion (McConnell et al., 2016).

SLIT signaling also regulates cadherins; SLIT-ROBO binding suppresses N-cadherin (CDH2)-dependent cell adhesion, via the formation of the ROBO-CDH2 *cis*-complex mediated by ABL (Rhee et al., 2007). SLIT-ROBO-ABL signaling then uncouples CDH2 from β-catenin (CTNNB1) and F-actin. Thus, the filopodial cell-adhesion machinery may be regulated by SLIT-ROBO-ENA-ABL signaling.

It has been documented that guidance cues modulate axon fasciculation (Tessier-Lavigne and Goodman, 1996; Chédotal, 2019). Fasciculation ensures precise control of follower axon extension and navigation to proper synaptic fields. SLIT-ROBO signaling enhances motor axon fasciculation (Jaworski and Tessier-Lavigne, 2012). Consistently, in spinal commissural neurons and cortical neurons, SLIT2 stimulation (30–40 min) promotes dynamic axon fasciculation and cell-cell interactions (Fig. 3C). SLIT-ROBO regulation of cell-cell contacts is reminiscent of protocadherin (PCDH17)-mediated, homophilic cell-cell interactions (Hayashi et al., 2014). Both systems may utilize ENA/VASP and WAVE to promote membrane protrusion and collective cell migration. Thus, repellents, together with CAMs, allow selective axon fasciculation and sorting. Fasciculation-based axon-sorting is important for the olfactory map development (see below).

### The semaphorin signaling pathways

More than 20 semaphorin (SEMA) members have been characterized and classified into secreted, transmembrane, and GPI-anchored types (Fig. 1) (Pasterkamp, 2012; Worzfeld and Offermanns, 2014). They are further categorized into eight classes based on their domain structures. Typical semaphorin receptors are neuropilins (NRPs) and plexins (PLXNs) (Fig. 4A–D). Semaphorins and plexins share the N-terminal Sema domain (Fig. 1). As signaling receptors, plexins can act alone or in combination with neuropilins or other co-receptors. Neuropilins and plexins associate with each other to form SEMA3 receptor complexes (Tamagnone et al., 1999). In vertebrates, nine plexin members have been identified and classified into four subfamilies (PLXNA–D). Vertebrates have two neuropilins, both containing short cytoplasmic domains and acting as binding subunits for SEMA3 and other ligands. Most membrane-tethered semaphorins are capable of binding to plexins directly, whereas secreted SEMA3s bind to the neuropilin-plexin complexes, with the exception of SEMA3E-PLXND1 signaling (not involving neuropilins; Gu et al., 2005).

Vascular endothelial growth factor A (VEGFA), a potent angiogenic factor, binds to neuropilins in endothelial and tumor cells (Soker et al., 1998). VEGFA binds to receptor complexes composed of VEGF receptors (VEGFRs) and neuropilins (Adams and Eichmann, 2010; Napolitano and Tamagnone, 2019). Neuropilins also interact with other receptors, including EGF receptor, insulin-like growth factor-1 receptor and β1-integrin, acting as multifunctional receptor components. Neuropilins modulate signal-transducing activities of partner co-receptors, possibly by controlling their intracellular trafficking (Napolitano and Tamagnone, 2019).

Several transmembrane semaphorins play immunoregulatory roles (Suzuki et al., 2008) and are called immune semaphorins (Suzuki et al., 2008). GPI-anchored SEMA7A on activated T cells binds to α1β1-integrin on macrophages, stimulating macrophages through immunological synapses (Suzuki et al., 2008; see also the disease section). In the nervous system, SEMA7A binds to β1-integrin, to promote axon growth and to PLXNC1, to regulate neurogenesis and synaptic plasticity (Fig. 4D) (Uesaka et al., 2014; Jongbloets et al., 2017; Inoue et al., 2021). Thus, semaphorins play diverse roles in various cell types, via distinct receptors. One unique feature of semaphorin signaling is that all plexins contain two GAP domains for small GTPases R-RAS and RAP1B/2A (Fig. 4A–D) (Oinuma et al., 2004).

MICAL (molecule interacting with CasL; aka, microtubule-associated monooxygenase, calponin and LIM domain-containing) is a plexin-binding protein that transduces signals from semaphorins to the actin cytoskeleton (Terman et al., 2002). MICALs contain an F-actin-interacting calponin homology domain and a flavoprotein monooxygenase domain, whose oxidoreductase activity is essential for semaphorin-mediated repulsion of dorsal root ganglion (DRG) axons. MICAL oxidizes two methionine residues of actin, thereby disassembling F-actin to cause axon repulsion upon semaphorin stimulation (Hung et al., 2011). Thus, semaphorin signaling incorporates redox control via MICAL.

The diversity of semaphorin pathways is also derived from catalytic or modulatory signal transducers that bind to neuropilins/plexins. Plexins also associate with receptor tyrosine kinases (RTKs) and cytoplasmic tyrosine kinases (Tamagnone et al., 1999). Cytoplasmic transducers of the CRMP (collapsin response mediator protein) family also participate in semaphorin signaling (Goshima et al., 1995) to regulate cytoskeletal dynamics. Thus, semaphorin pathways regulate different processes using a wide range of signal transducers, including Ras/Rap GTPases, redox enzymes, protein kinases, and CRMPs.

### The ephrin signaling pathways

EPH (erythropoietin-producing human hepatocellular) receptors constitute the largest subfamily of RTKs. Although EPHs were initially identified as orphan receptors, many membrane-tethered ligands, called ephrins (EFNs), have been characterized (Kania and Klein, 2016). EFN-EPH signaling mainly mediates short-range cell-cell communications, including cell contact-mediated repulsion or adhesion, in axon mapping and fasciculation, angiogenesis, and tumorigenesis (Adams and Eichmann, 2010; Kania and Klein, 2016). Ephrins are classified into GPI-anchored ephrin-As (EFNAs) and transmembrane ephrin-Bs (EFNBs). Correspondingly, EPH receptors are classified into two subclasses, EPHAs and EPHBs, based on their binding affinity and structural similarity (Fig. 1) (Kania and Klein, 2016). EPHs are unique among the guidance receptors in that they contain kinase domains. EFNAs and EFNBs bind to EPHAs and EPHBs, respectively, with some exceptions (e.g. EFNB3–EPHA4 interactions in corticospinal axon guidance; Iwasato et al., 2007).

Importantly, ephrin pathways utilize forward, reverse, and bidirectional signaling modes. EFN-EPH signaling regulates the shape and motility of EFN-expressing cells and EPH-expressing cells upon their contacts. Such EFN–EPH signaling prevents cell mixing, thereby forming the compartmental boundaries during morphogenesis. For example, during convergent extension in vertebrate embryos, multiple EFN–EPH pairs drive cell sorting by suppressing cadherin-mediated adhesion. EFN–EPH interactions lead to heterotypic cell repulsion by reducing the stability of cell contacts, whereas EFN–EFN or EPH–EPH interactions cause homotypic cell aggregation (Kindberg et al., 2021).

EFN–EPH binding activates the EPH kinase domain, triggering EPH autophosphorylation (Kania and Klein, 2016). In EPHB–EFNB reverse signaling, EFNB-ICD is phosphorylated by associated SRC family kinases. Activated EPHs phosphorylate many other protein kinases/phosphatases and adaptors. Asymmetric activation or inactivation of effectors in EFN- or EPH-expressing cells leads to repulsive cell-sorting (Jørgensen et al., 2009). Several EFN- and EPH-specific RhoGEFs/GAPs have been identified. For example, EPHA4 interacts with RHOA-GEF ephexin1 (NGEF), RAC1-GEF VAV2, and RAC1-GAP CHN1 (Fig. 4E) (Kania and Klein, 2016). Thus, dynamic regulation of RhoGTPase activity mediates EFN-EPH-induced cell repulsion and possibly other EPH-regulated processes.

### Non-canonical neuronal guidance genes

Morphogens (such as sonic hedgehog [SHH], WNTs, bone morphogenetic proteins [BMPs]), chemokines, and a subset of growth factors can act as non-canonical attractants or repellents for axons and neurons (Chédotal, 2019; Zou, 2020a, b). Neural tube patterning is induced by SHH released from the notochord. Following the SHH-dependent floorplate (FP) differentiation, FP cells produce SHH and organize neuronal differentiation within the neural tube (Jessell, 2000). FP-derived SHH also acts as a chemoattractant that directs commissural axon growth to the midline, together with netrin-1, but is switched to be sensed as a chemorepellent for longitudinal axon growth upon midline crossing (Bourikas et al., 2005; Wu et al., 2019).

WNT pathways regulate planar cell polarity (PCP) to polarize growth cones for pathfinding (Zou, 2020a). WNT4 is expressed in a rostral-caudal decreasing gradient in the FP of developing spinal cords. WNT4-Frizzled3 (FZD3) receptor signaling directs commissural axons to turn rostrally after midline crossing (Lyuksyutova et al., 2003). Importantly, the WNT-PCP machinery also facilitates axon-axon communications and is regulated by leucine-rich repeat kinase-2 (LRRK2, encoded by *PARK8*, a causal gene for familial Parkinson’s disease)-mediated FZD3 phosphorylation (Onishi et al., 2020). LRRK2 dysfunction compromises axon-axon communications of commissural neurons and midbrain dopamine neurons, causing axon pathfinding errors. In addition, WNT3-RYK receptor signaling, together with EFNB–EPHB signaling, regulates dorsal-ventral retinotopic mapping (see below; Schmitt et al., 2006). Thus, the WNT pathways ensure the fidelity of axonal decisions.

The chemokine SDF-1 (CXCL12) not only directs immune-cell chemotaxis but also acts as a chemoattractant for various neuronal types (Zou et al., 1998). Interestingly, CXCL12-CXCR4 receptor signaling does not attract spinal motor neurons, rather may attenuate repulsive responses, when CXCL12 and repellents are sensed simultaneously (Lieberam et al., 2005).

### RTN4R-mediated axonal growth and synaptogenesis

After neural damage, RTN4 (reticulon-4) receptors (RTN4R, also called NOGO receptor 1 [NGR1]) and oligodendrocyte-derived RTN4/NOGO proteins inhibit neuroregeneration (Fig. 1; discussed later). However, under physiological conditions, RTN4Rs act as “ligands” for brain-specific angiogenic inhibitors (BAIs), adhesion-GPCRs, and regulate axon/dendrite growth, dendritic arborization, and synaptogenesis, thereby playing essential roles in synaptic network formation (Wang et al., 2021).

## Mechanisms underlying neuronal guidance

Many critical steps in neural development are coordinated by combinatorial regulation of neuronal guidance signaling. Here we discuss the major mechanisms in several representative processes.

### Midline axon guidance, a model system

Along the way to distant synaptic targets, growing axons use a series of intermediate targets as guideposts. Axons change their responsiveness to multiple guidance cues at the right place and time. Midline axon guidance has served as a model for dissecting molecular mechanisms underlying spatiotemporal control of axon pathfinding. Many commissural neurons are born dorsally, extend their axons ventrally in the neural tube, cross the midline, and project contralaterally, whereas many other classes of axons never cross the midline and project ipsilaterally. Floorplate (FP) cells at the midline act as a representative guidepost and produce both attractants and repellents, such as netrins and SLITs, for commissural and ipsilaterally projecting axons, although this simple picture has recently been challenged (Dominici et al., 2017; Chédotal, 2019).

What mechanisms make axons decide to cross the midline or not? This rudimentary question is not fully understood. Commissural axons initially respond to attractants, but not to repellents, and grow ventrally. However, upon reaching the midline, axons lose their responsiveness to the attractants and acquire responsiveness to repellents. Thus, commissural axons recognize the midline as an unfavorable area. After crossing and exiting the midline, axons never re-cross it. Below, we focus on three key problems for discussion.

1) How are commissural axons guided toward the midline by attractants?

Netrin-1 is produced by not only FP cells but also neural progenitors in the ventricular zone (VZ) (Dominici et al., 2017). It has long been considered that a concentration gradient of FP-derived netrin-1 is required and sufficient for commissural axon attraction to the midline (Fig. 2C, left). However, analyses of conditional knockout mice in which netrin-1 is genetically removed from cells of either FP or VZ revealed the following: 1) FP-derived netrin-1 is dispensable for commissural axons to reach the midline; 2) VZ-derived netrin-1 is required for axon midline crossing, at least in the hindbrain and 3) netrin-1 can act as a short-range, contact-mediated cue (Dominici et al., 2017). VZ neural progenitors extend radial processes toward the pial surface. Netrin-1 is transported and distributed along these radial processes and presented to commissural axons (Fig. 2C, right). Subsequent studies suggest that FP- and VZ-derived netrin-1 cooperatively contributes to commissural axon guidance, because few commissural axons cross the midline in mice with simultaneous deletions of netrin-1 in FP and VZ cells (Moreno-Bravo et al., 2019). Thus, the current consensus is that both VZ-derived, membrane-tethered netrin-1 and FP-derived, diffusible netrin-1, together with SHH, synergistically guide commissural axons to the midline (Moreno-Bravo et al., 2019; Wu et al., 2019). It is unclear whether diffusible netrin-1 and immobilized netrin-1 activate distinct signaling cascades.

2). How do commissural axons turn off responses to attractants at the midline?

To control sensitivity to multiple cues, one repetitively used strategy is hierarchical silencing of receptors. Upon reaching the midline, commissural axons sense SLIT and lose netrin-1 responsiveness through ROBO1-mediated silencing of DCC function (Shirasaki et al., 1998; Stein and Tessier-Lavigne, 2001). Presenilin-1 (PSEN1), the catalytic subunit of the γ-secretase complex, proteolytically cleaves DCC in motor axons (Bai et al., 2011). Upon matrix metalloproteinase-mediated cleavage of DCC at the extracellular domain (ECD), γ-secretase cleaves the “DCC stub” (composed of the transmembrane and intracellular domains) to release the DCC-ICD. In the absence of PSEN1, accumulated DCC stubs block SLIT-ROBO1-mediated DCC silencing and maintain netrin-1 signaling competency. Thus, γ-secretase is crucial for suppressing netrin-1 attraction of motor axons and possibly also for commissural axon midline crossing.

Conversely, DCC suppresses Robo before commissural axons reach the midline in *Drosophila*. DCC mediates netrin attraction; and simultaneously, the γ-secretase-processed DCC-ICD is transported to the nucleus and transcriptionally activates *Comm*, suppressing Robo function (Neuhaus-Follini et al., 2015). Thus, hierarchical receptor regulations contribute to spatiotemporal control of attraction and repulsion.

3). How do commissural axons acquire SLIT responsiveness upon midline crossing?

In vertebrates, FP cells secrete SLIT1–3 and SEMA3B/3F, which are sensed by ROBO1/2, PLXNA1, and NRP2 expressed in commissural axons. In *Drosophila*, midline glial cells secrete Slit. Upon reaching and crossing the midline, commissural axons acquire responsiveness to the repellents. This timing is critical for axons to successfully cross the midline (Zou et al., 2000). Comm (in *Drosophila*) and ROBO3 (in vertebrates) suppress SLIT responsiveness in pre-crossing commissural axons (Sabatier et al., 2004; Keleman et al., 2005; Chen et al., 2008). Comm prevents Robo presentation on their axon surface by sorting Robo to late endosomes. ROBO3 has two isoforms with different C-terminal tails. ROBO3.1 suppresses SLIT response of pre-crossing commissural axons, whereas ROBO3.2 promotes SLIT response (Chen et al., 2008). It remains unclear how ROBO3.1 suppresses SLIT response of commissural axons.

SLIT-ROBO1 signaling activates the small GTPase ARF6, a regulator of endocytosis and recycling (Kinoshita-Kawada et al., 2019). ARF6 is required for commissural axon midline crossing. Distinct cytohesin ArfGEFs, CYTH2, and CYTH1, negatively and positively regulate SLIT responsiveness before and after midline crossing, respectively. These mechanisms are required for self-enhancement of SLIT response of commissural axons. The “memory” of acquisition of responsiveness to a guidance cue may be maintained by the self-sensitization, enabling the irreversible path selection. Consistently, live-imaging analyses revealed sequential sorting of NRP2, PLXNA1, ROBO1, and ROBO2 to the growth cone surface during midline crossing, which sensitizes growth cones to SLIT and SEMA3B/3F (Pignata et al., 2019).

Vertebrate Comm homologues, PRRG4, and NEDD4 (E3 ubiquitin ligase)-interacting proteins (NDFIP1/2), were recently found (Justice et al., 2017; Gorla et al., 2019). Before axons reach the midline, NDFIP1/2 sort ROBO1 to late endosomes, and direct NEDD4-mediated ROBO1 ubiquitination and degradation (Gorla et al., 2019). Interestingly, the deubiquitinase USP33 is essential for SLIT responsiveness of commissural axons and midline crossing (Yuasa-Kawada et al., 2009). The spatiotemporal balance between ubiquitination and deubiquitination of ROBO1 may achieve fine control of SLIT responsiveness of commissural axons. Thus, multi-layered mechanisms regulate ROBO dynamics to ensure that commissural axons acquire SLIT responsiveness after midline crossing.

### Neurogenesis and apoptosis

Various guidance cues have been reported to regulate neurogenesis and apoptosis. SLIT-ROBO signaling modulates neurogenesis by controlling generation, self-renewal and maintenance of neural progenitors (reviewed by Llinares-Benadero and Borrell, 2019). *Robo1*/*2* knockouts result in reduced mitosis of apical progenitors (Borrell et al., 2012). ROBO collaborates with NOTCH, and controls asymmetric cell division and cell-fate determination (Borrell et al., 2012). The outputs of SLIT-ROBO and DLL1 (Delta-like 1)-NOTCH signaling determine the mode of direct neurogenesis from radial glial cells versus indirect neurogenesis via intermediate progenitor cells (Cárdenas et al., 2018).

In the hippocampal dentate gyrus (DG), where adult neurogenesis occurs, SEMA7A on granule cells inhibits proliferation of adjacent neural progenitors, via PLXNC1. During differentiation of adult-born DG granule cells, SEMA7A stimulates dendritic spine development via β1-integrin (Jongbloets et al., 2017). Thus, SEMA7A regulates neurogenesis and subsequent maturation of adult-born neurons, via distinct receptors. Ephrins also regulate neurogenesis by activating transient waves of apoptosis of neural progenitors, thereby affecting brain size (Depaepe et al., 2005; Kellermeyer et al., 2018). Thus, SEMA and ephrin signaling pathways may contribute to neural circuit patterning by regulating not only axonal guidance but also neurogenesis and apoptosis.

### Substantia nigra development

**Figure 5.**
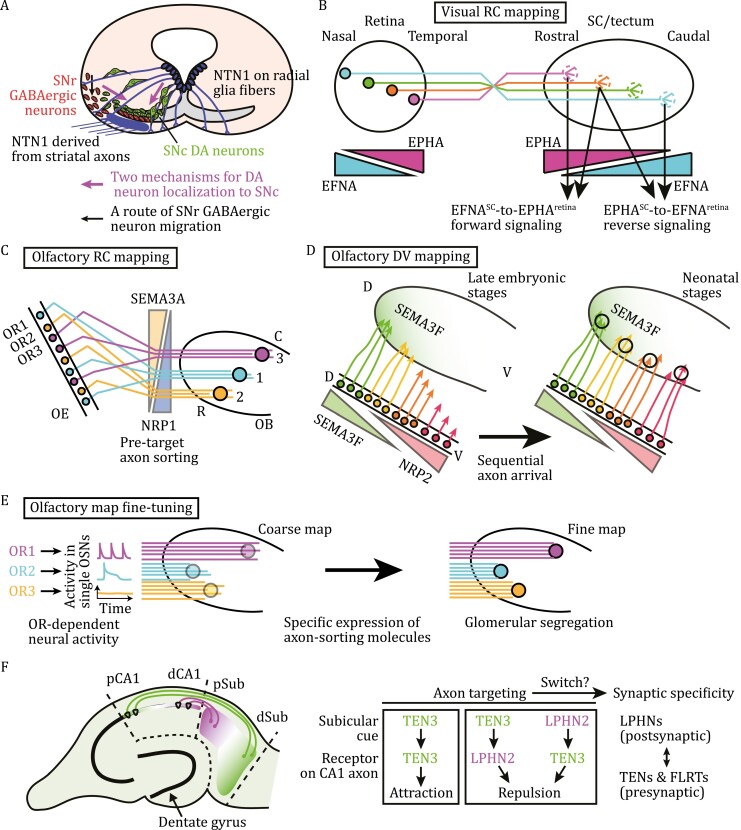
Neuronal migration into the substantia nigra and the formation of representative neural maps. (A) A schematic illustration of two routes of neuronal migration guided by NTN1. Migration of DCC-expressing dopamine (DA) neurons (green cells) into the SNc is guided by NTN1 presented along VZ radial glial fibers (purple lines), whereas migration of DSCAM-expressing GABAergic neurons (red cells) into the SNr is directed by NTN1 axonally delivered from the forebrain striatum (purple). GABAergic neurons further restrict tangentially migrating DA neurons to the SNc. (B) The retinocollicular (or retinotectal in lower vertebrates) projection. Two countergradients (EFNA and EPHA) contribute to the establishment of the retinotopy on the superior colliculus (SC) along the rostral-caudal (RC) axis by triggering EFNA-EPHA forward and reverse signaling. Temporal retinal axons project to the rostral SC, whereas nasal axons project to the caudal SC, thereby establishing a coarse retinocollicular map. A mature continuous map is formed after refinements. (C, D) The projection map formation of axons of olfactory sensory neurons (OSNs) on the olfactory epithelium (OE) to the glomerulus in the olfactory bulb (OB). During early phases of development, SEMA3A-NRP1 signaling regulates pre-target axonal sorting within axon bundles along the RC axis (C), whereas SEMA3F-NRP2 signaling controls the sequential arrival of OSN axons at the OB along the dorsal-ventral (DV) axis (D), thereby generating a coarse map. Note that, along the RC axis, odorant receptor (OR)-dependent spontaneous cAMP signaling specifies expression levels of SEMA3A and NRP1 in OSNs and their axon projection sites in the OB. (E) OR-directed, structured patterns of spontaneous neural activity fine-tune olfactory axon glomerular projections by inducing specific sets of axon-sorting molecules, to form a discrete map. (F) The hippocampal networks are generated by complementary gradients of TEN3 and LPHN2 in the hippocampal CA1 and subiculum (Sub): TEN3-TEN3 homophilic attraction and TEN3-LPHN2 reciprocal repulsion drives axon targeting. The TEN-FLRT-LPHN transsynaptic supercomplex then confers the synaptic specificity. d: distal; p: proximal.

The substantia nigra (SN) is a midbrain nucleus critical for motor control and reward functions. Made of two portions, substantia nigra pars compacta (SNc) and substantia nigra pars reticulata (SNr), the SN contains multiple types of neurons, including dopamine (DA) neurons that regulate voluntary movement. Molecular mechanisms underlying the SN subdivision during development are not fully understood. There are two routes for neuronal migration to the SN (Fig. 5A) (Brignani et al., 2020). First, VZ radial glia-derived netrin-1 guides ventral migration of populations of DCC-expressing DA neurons into the SNc. Second, netrin-1, produced at a distance by forebrain neurons, is delivered along axons to a ventral midbrain region and directs migration of DSCAM-expressing GABAergic neurons to the SNr. The SNr-directed movements of GABAergic neurons gradually exclude tangentially migrating DA neurons from the SNr, restricting them to the SNc (Brignani et al., 2020). As discussed for midline axon guidance, relocation of guidance cues from original sites of synthesis provides a major strategy for directing axons/neurons to distant targets (Fig. 2B and 2C).

### Establishing neural connectivity

Upon reaching their target areas, axons choose appropriately positioned synaptic partners. The regional specificity (topography) and laminar/subcellular specificity of synaptic connections are two major features of neural architecture. Sensory projection maps have been studied to understand molecular mechanisms establishing neural topography. Axon targeting initially generates a coarse map in the brain, followed by refinements to form a fine map (Fig. 5B–E) (Luo, 2021). Both neuronal guidance signaling and spontaneous neural activities contribute to neural map formation. Neural maps are further fine-tuned by experience throughout life. Below, we discuss several molecular mechanisms by which neuronal guidance signaling coordinates the fomation of neural maps.

#### Visual and olfactory topographic mapping

In many axon projections, the neighbor-to-neighbor relationship of projection neurons is preserved on their target fields, establishing continuous topographic maps, represented by the retinotopy of the visual system.

The optic tectum (superior colliculus [SC], in mammals) is a conserved midbrain nucleus that is critical for receiving retinal inputs and directing the movement of eyes and the body toward visual cues. After retinal axons reach the rosral SC/tectum, they extend caudally on the surface layer, then entering the retinorecipient layers. Axons initially overshoot their correct termination zones (TZs), forming interstitial branches. Incorrectly extended branches are eliminated during refinements, leading to map maturation. Earlier studies revealed that the temporal-to-nasal decreasing gradient of EPHA3 (in chickens; EPHA5/A6 in mice) in retinal ganglion cells (RGCs), together with the rostral-to-caudal increasing gradient of EFNA2/A5 in the SC/tectum, act as the chemical labels predicted by Sperry (Fig. 5B) (Drescher et al., 1995; Cheng et al., 1995). Although knockout studies support that EFNA^SC^-to-EPHA^retina^ signaling coordinates topographic retinocollicular mapping (Feldheim et al., 2000), several distinct mechanisms have also been proposed.

Strikingly, the countergradients of EFNAs and EPHAs along the rostral-caudal (RC) axis in both the retina and SC/tectum (“uphill” and “downhill” gradients, as shown in Fig. 5B) also contribute to retinotopic map development. Interestingly, both EFNAs and EPH receptors are colocalized on RGC axons. The *cis*-interacting EFNA^retina^-to-EPHA^retina^ module downregulates responsiveness of EPHA^high^ (EFNA^low^)-epxressing temporal retinal axons to EFNA^SC^ (Hornberger et al., 1999). This module may contribute to fine control of EFN responsiveness of RGC axons. Furthermore, EFNA^high^ (EPHA^low^)-expressing nasal axons are repelled by high-level EPHA in the rostral SC and pushed forward to the caudal SC, through reverse signaling (EPHA^SC^-to-EFNA^retina^ module) (Fig. 5B, right) (Rashid et al., 2005). This mechanism explains how nasal axons project to the caudal SC. Collectively, repulsive EFNA^SC^-to-EPHA^retina^ forward signaling and repulsive EPHA^SC^-to-EFNA^retina^ reverse signaling organize the RC mapping.

On the other hand, retinotectal/retinocollicular mapping along the dorsal–ventral (DV) axis is coordinated by two counterbalanced signaling activities: attractive EFNB-EPHB signaling (Mann et al., 2002) and WNT3-RYK receptor signaling (Schmitt et al., 2006). In the latter regulation, WNT3 induces biphasic responses. WNT3 binds to RYK, repelling ventral RGC axons. However, at lower concentrations, WNT3 binds to FZD5 receptor, attracting dorsal axons. EFNB1 and WNT3 are expressed in dorsal-ventral decreasing gradients in the SC/tectum. Collaboratively, EFNB and WNT signaling pathways organize retinotopic DV mapping.

Odor information is transmitted from olfactory sensory neurons (OSNs) in the olfactory epithelium (OE) to their target neurons in the glomerulus of the olfactory bulb (OB). Remarkably, the olfactory system exhibits a discrete topographic map (Fig. 5C–E). In rodents, OSNs sense odors through approximately 1000 types of odorant receptors (ORs), which constitute the largest subfamily of GPCRs (Buck and Axel, 1991; Mori and Sakano, 2011). Each OSN expresses only one functional OR gene. Although OSNs expressing a given OR type appear to be randomly distributed in the OE, the OSN distribution actually adapts a zone-specific manner, with their axons converging onto a specific glomerulus in the ipsilateral OB. Thus, odor information is represented as specific combinations of activated glomeruli and sent to the olfactory cortex (Mori and Sakano, 2011). A series of studies have demonstrated that repellents, such as SEMA3A and SEMA3F, and their receptors, together with various CAMs, direct selective fasciculation and sorting of OSN axons to reach the appropriate glomeruli (reviewed by Lodovichi, 2021).

Olfactory mapping requires two independent axon-targeting mechanisms along RC and DV axes. Along the RC axis, OR-derived cAMP signals determine the OSN axon projection sites in the OB. Such OR-derived cAMP signals are evoked by interactions of spontaneously active ORs with trimeric Gs proteins. cAMP signals are decoded into the graded expression of SEMA3A receptors, NRP1and PLXNA1, in OSNs (Imai et al., 2006; Nakashima et al., 2013). Consistently, expression of a constitutively active mutant of cAMP response element-binding protein (CREB), a transcription regulator, altered axon projection sites in the OB along the RC axis (Imai et al., 2006). Importantly, complementary gradients of SEMA3A and NRP1 in OSNs, directed by the OR-derived cAMP signals, instruct axon sorting within OSN axon bundles before reaching the OB, so that the same OR-expressing axons converge onto particular glomeruli (Fig. 5C) (Imai et al., 2009).

On the other hand, the DV targeting is dependent on the complementary gradients of SEMA3F and NRP2 in OSNs, but not controlled by OR-derived signals (Takeuchi et al., 2010). Positional information of OSNs within the OE dictates the specific OR gene expression and the levels of SEMA3F and NRP2 in OSNs. Thus, axons of particular OR-expressing OSNs appear to be targeted to specific glomeruli also along the DV axis. In the OB, earlier-arriving OSN axons secrete higher levels of SEMA3F, which facilitate axon sorting before glomerular targeting by repelling the later-arriving NRP2-expressing OSN axons. This mechanism enables the sorting of sequentially arriving axons to distinct glomeruli (Fig. 5D) (Takeuchi et al., 2010). Therefore, neuronal guidance signaling controls selective axon fasciculation and sorting along both RC and DV axes, thereby organizing a coarse olfactory map in the OB.

#### The hippocampal system

The hippocampal formation, a structure critical for learning and memory, contains several subregions: the hippocampus proper or cornu ammonis (CA1–3), the dentate gyrus and subiculum. Hippocampal networks are topographically wired: axons from the proximal CA1 project to the distal subiculum, whereas distal CA1 axons project to the proximal subiculum (Fig. 5F). Sperry’s chemoaffinity hypothesis (1963) seems to apply also to hippocampal networks. Teneurin3 (TEN3) is expressed in different hippocampal regions, including proximal CA1 and distal subiculum, and in the medial entorhinal cortex (not shown) (Berns et al., 2018).

In many cases, guidance cues and receptors are heterophilic, as in EFN-EPH signaling. In contrast, TEN3 acts as a contact-mediated, homophilic attractant for targeting proximal CA1 axons to the distal subiculum (Berns et al., 2018). In the hippocampal networks, TEN3 and Latrophilin2 (LPHN2, a synaptic adhesion-GPCR that binds to TEN3) are expressed in complementarily graded fashions (Fig. 5F). Strikingly, TEN3 and LPHN2 mutually act as contact-mediated repellents (reciprocal repulsion) in establishing the CA1-to-subiculum connections (Pederick et al., 2021). TEN3^**+**^ proximal CA1 axons are repelled by proximal subicular LPHN2, whereas LPHN2^**+**^ distal CA1 axons are repelled by distal subicular TEN3 (Fig. 5F). Thus, TEN3-TEN3-mediated homophilic attraction and TEN3-LPHN2-mediated reciprocal repulsion establish the "TEN3^**+**^ axon-to-TEN3^**+**^ target" and "LPHN2^+^ axon-to-LPHN2^+^ target" rules in the hippocampus-subiculum projection.

On the other hand, TENs and LPHNs, along with FLRTs, also mediate synaptic partner choice (Sando et al., 2019). Since synaptogenesis is an intrinsically asymmetric process, during which presynaptic endings and postsynaptic specializations differentiate (Südhof, 2021), it is unclear how asymmetric synaptic structures emerge upon TEN3-TEN3 homophilic binding (Fig. 5F). Furthermore, it remains unknown how TEN3-TEN3-mediated attractive axon targeting is switched to the TEN-FLRT-LPHN-directed synapse formation.

#### Laminar/subcellular synapse specificity

Upon reaching topographically matched sites, axons choose target neurons located in appropriate laminae and form synapses with subcellular-level specificity. Multiple CAMs participate in these transsynaptic cell-recognition processes; and combinatorial codes of such CAMs may determine synaptic specificity (Sanes and Zipursky, 2020; Südhof, 2021). Although detailed mechanisms remain to be understood, neuronal guidance cues seem to enhance synaptic specificity. For example, semaphorins are involved in sharpening axonal/dendritic fields of individual neurons. Repulsive signaling between transmembrane SEMA6A and PLXNA4 (and/or PLXNA2) restricts axon projection, neurite arborization and synapse formation in the specific laminae (Matsuoka et al., 2011; Sun et al., 2013). In the vertebrate retina, the directional motion of visual cues is processed through synaptic connections among bipolar cells, starburst amacrine cells (SACs), and direction-selective RGCs. SEMA6A is expressed in On-type, but not Off-type, SACs, whereas PLXNA2 is expressed in both On- and Off-type SACs. Repulsive SEMA6A-PLXNA2 signaling achieves sublaminar segregation of On- and Off-type SAC dendrites and then symmetric dendritic arborization in On-type SACs (Sun et al., 2013). These mechanisms underlie the establishment of the motion-detecting retinal circuitry. In addition, the majority of excitatory synapses are formed on dendritic spines, whose dynamics is correlated with synaptic plasticity. Various guidance cues may modulate synaptic formation and plasticity by affecting dynamics of the dendritic spines (Dong et al., 2015).

#### Fine-tuning neural wiring

The initial coarse neural map undergoes remodeling to gain precise synapse specificity (Luo, 2021). Map refinements involve neural activity-dependent and -independent processes. Spontaneous activity occurs before birth and during early postnatal periods to drive map refinement (McLaughlin et al., 2003), and gradually switches to experience-based activity. In the developing visual system, spatially moving, correlated patterns of spontaneous firing of RGC neurons propagate across the retina before eye-opening. Such retinal waves exihibit a propagation bias reflecting the future optic flow generated by forward motion simulation (Ge et al., 2021). Coarse retinotopic maps generated by EFN-EPH signaling are extensively remodeled by retinal wave propagation. Deleting both EFNAs and retinal waves, but not either alone, disrupts retinotopic map formation (Cang et al., 2008). Thus, EFNA-EPHA signaling and neural activity synergistically contribute to the retinotopy. These common mechanisms generate retinotopic maps not only in the superior colliculus, but also in the thalamus and cerebral cortex.

Although it remains elusive how neural activity collaborates with neuronal guidance signaling to establish topographic maps, abundant evidence indicates that neural activity modulates guidance signaling. In the olfactory system, odorant receptors (ORs) determine structured patterns (e.g. short or prolonged burst) of spontaneous firing in OSNs (Fig. 5E). OR-dependent activities induce expression of specific sets of axon-sorting molecules, such as CAMs and repellents (e.g. SEMA7A), and drive glomerular segregation of OSN axons (Nakashima et al., 2019; Inoue et al., 2021). Thus, spontaneous signals and odor signals received after birth may regulate repulsive and adhesive signaling genes to establish a discrete olfactory map.

Furthermore, neural activity modulates the synaptic release of neurotransmitters, which bind to cognate GPCRs to activate adenylate cyclases and alter the cAMP/cGMP balance in postsynaptic neurons (Hempel et al., 1996). Consistently, neuronal responses to guidance cues, including ephrins and semaphorins, are regulated by neural activity in Ca^2+^- and cAMP-dependent manners (Ming et al., 2001; Carrillo et al., 2010). For example, neural activity-triggered presynaptic calium influx through voltage-gated Ca^2+^ channels enhance retrograde semaphorin signaling, thereby reducing ectopic synaptic contacts in *Drosophila* (Carrillo et al., 2010). Retrograde semaphorin signaling is also essential for elimination of redundant synapses between climbing fibers and Purkinje cells in the mouse cerebellum. SEMA7A promotes the synapse elimination, via presynaptic PLXNC1 and β1-integrin (Fig. 4D), whereas SEMA3A exhibits the opposite effects (Uesaka et al., 2014). Thus, neural activity, coupled with guidance signaling, modulates elimination of off-target synaptic contacts, multi-scale pruning of branches, and possibly, preferential elaboration of appropriate arbors.

Although activity-independent mechanisms underlying postsynaptic dendrite patterning remain unclear, evidence in *C. elegans* has shed light on an netrin-mediated mechanism (Ramirez-Suarez et al., 2019). Netrin is captured by a neuronal ECM protein, Papilin, and presented to axons and dendrites, to pattern lateral nerve tracts. This is another example of using the cue-presentation system (see Fig. 2A).

#### Homeostatic synaptic scaling

Homeostatic scaling is a synaptic plasticity mechanism that regulates neuronal firing (Turrigiano, 2017). At excitatory synapses of cortical neurons, NRP2/PLXNA3 complexes interact with postsynaptic AMPA-type glutamate receptors (AMPARs), and regulate homeostatic downscaling of cell-surface AMPARs in response to SEMA3F (Wang et al., 2017). SEMA3F is secreted by cortical neurons in a neural activity-depednent manner, although whether SEMA3F is released pre- or post-synaptically remains unclear. This reveals a negative feedback mechanism to suppress excessive neural activity. In *Drosophila*, Sema2b acts as a retrograde signal and binds to presynaptic PlexB for regulating neurotransmitter release at neuromuscular junctions (Orr et al., 2017). Thus, semaphorin signaling constitutes an evelutionally conserved mechanism for homeostatic scaling.

Many neuronal guidance cues, such as netrins and SLITs, their receptors and downstream components, are expressed in not only developing but also adult brains. Therefore, it is likely that their roles may not be limited to “guidance” function during development. Further studies will be necessary to elucidate molecular mechanisms by which guidance cues control synaptic remodeling and plasticity in adulthood.

## Neuronal guidance genes in organogenesis and homeostasis

Neuronal guidance genes regulate many processes outside the nervous system, including angiogenesis, immune cell migration/function, and various aspects of organogenesis (Wu et al., 2001; Lu et al., 2004; Wilson et al., 2006; Branchfield et al., 2016). Molecular mechanisms by which neuronal guidance genes regulate the formation of various tissues and organs have begun to emerge. We focus on a few model systems to show multifaceted roles of guidance genes in organogenesis and homeostasis.

The key roles of different neuronal guidance genes in regulating cell-cell communications have been recently reviewed (e.g. Beamish et al., 2018). In addition, tunnelling nanotubes (TNTs) have been attracting attention as a strategy for cell–cell communications and organelle transfer (Wang et al., 2021 and references within). MICAL2 variants suppress TNT formation and mitochondrial transfer by inhibiting RHOT2 (MIRO2), a RhoGTPase (Wang et al., 2021). It is possible that other neuronal guidance genes may regulate TNT-mediated organelle transfer. Macrophages survey their environments via macropinocytosis, but also can trigger inflammatory responses. Recent evidence suggests that SLIT2 suppress macrophage macropinocytosis and pathological inflammation (Bhosle et al., 2020). Hematopoietic stem cells (HSCs) generate all blood cell types. HSC dormancy ensures long-term maintenance of their self-renewal potentials. Niche-derived netrin-1 confers the dormancy to NEO1-expressing HSCs (Renders et al., 2021). These examples show versatile roles of neuronal guidance genes in non-neuronal cell communications.

### The cardiovascular system

Several neuronal guidance signaling pathways have been studied in the cardiovascular system. *EfnB2* and its receptor, *EphB4*, are expressed in arterial and venous endothelium, respectively, and regulate angiogenesis of arteries and veins (Wang et al., 1998). Furthermore, SEMA3-NRP1-PLXND1 and SLIT-ROBO1 pathways regulate heart development. Knockouts for *Sema3*, *Nrp1*, or *PlxnD1* and *Robo1* mutant mice show ventricular septal defects (VSDs) and outflow tract defects, suggesting dysfunction of these guidance pathways may contribute to congenital heart disease (CHD) (Gu et al., 2003; Gitler et al., 2004; Kruszka et al., 2017). These studies support the role of guidance gene signaling in coordinating cardiovascular morphogenesis.

**Figure 6.**
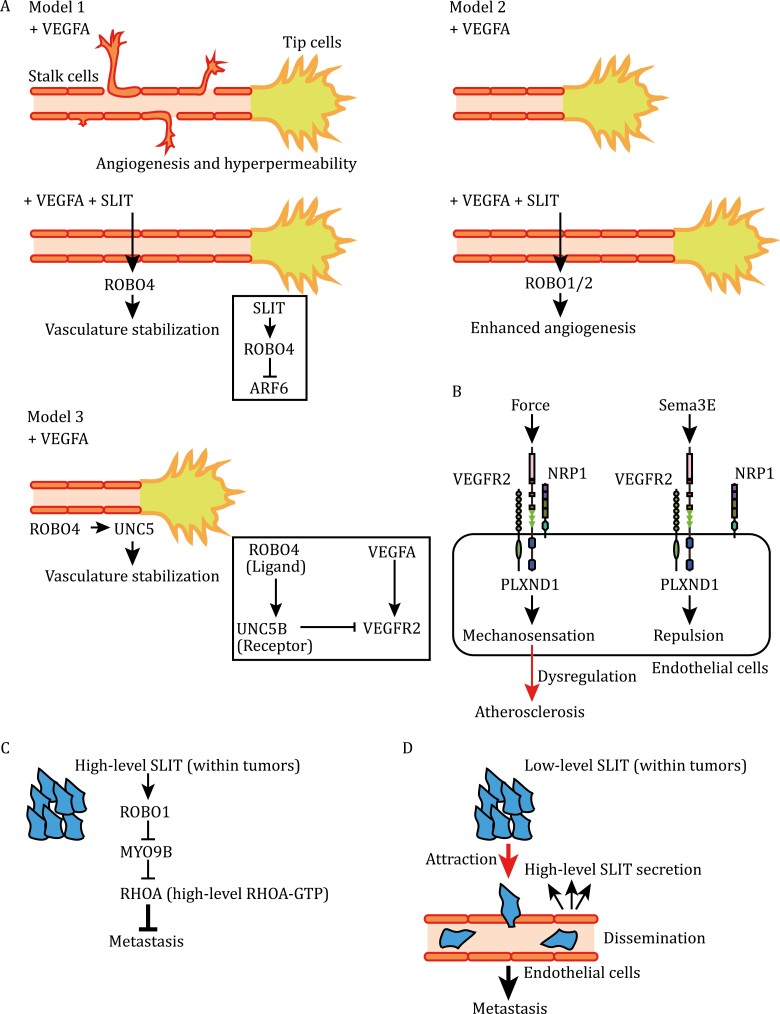
Neuronal guidance genes in regulating angiogenesis and cancer metastasis. (A) Models proposed for functions of guidance cues in angiogenesis. In model 1, in response to SLIT, ROBO4 stablizes the vasculature by suppressing ARF6 activity and thus VEGFA-induced angiogenesis and hyperpermeability. In model 2, SLIT activates angiogenic processes via ROBO1/2. In model 3, upon ROBO4-UNC5 binding, UNC5 suppresses VEGFA-induced angiogenesis and hyperpermeability. (B) Roles of PLXND1 in mechanosensation and in SEMA3E-induced repulsion in endothelial cells. PLXND1 forms mechanosensory or SEMA3E receptor complexes by differentially interacting with NRP1 and VEGFR2. (C) Intratumoral SLIT-ROBO1-MYO9B signaling suppresses cancer metastasis. (D) SLIT acts as a chemoattractant for tumor cells, when endothelial cells express SLIT at higher levels than tumor cells. SLIT-ROBO1 signaling may thus promote cancer metastasis.

The nervous and vascular systems share common extracellular cues and receptors for wiring of nerves and blood vessels (Carmeliet and Tessier-Lavigne, 2005). Tip cells, motile endothelial cells at the distal ends of vessel sprouts, equipped with filopodia, direct sprout extension by sensing VEGFA and neuronal guidance cues (Fig. 6A) (Adams and Eichmann, 2010). Signaling pathways of VEGFA and guidance cues communicate with each other, mediating neurovascular interactions that achieve alignments of blood vessels with axon fibers. Below, we discuss several guidance signaling mechanisms in angiogenesis.

The role of netrin in vascular growth remains controversial, as it was reported either as an inhibitor (Lu et al., 2004) or a promoter (Wilson et al., 2006). ROBO4 is highly expressed in endothelial cells. In response to SLIT, ROBO4 signaling inhibits ARF6 activity and stabilizes the vasculature by suppressing VEGFA-induced angiogenesis and vascular leakage (Fig. 6A; Model 1) (Jones et al., 2009). However, it was also reported that SLIT does not bind to ROBO4 but promotes angiogenesis via ROBO1/2 (Fig. 6A; Model 2) (Wang et al., 2003; Koch et al., 2011; Rama et al., 2015). It remains controversial whether ROBO4 acts as a SLIT receptor and whether SLIT primarily acts as an anti- or pro-angiogenic factor. Unexpectedly, ROBO4 binds to UNC5B in *trans*, acting mutually as ligands. Their interactions maintain vascular integrity by activating UNC5B, which inhibits VEGFA-induced angiogenesis (Fig. 6A; Model 3) (Koch et al., 2011). Furthermore, ROBO4 suppresses breast cancer growth and metastasis by reducing tumoral angiogenesis and vascular permeability (Zhao et al., 2016). Taken together, ROBO4 increases vascular stability. It is unclear how netrins and ROBO4 exert different effects on endothelial cells, through UNC5B. Clearly, more work is necessary to fully understand vascular netrin and SLIT signaling.

Although SEMA3A and VEGFA bind to NRP1 in endotheial cells (Soker et al., 1998), VEGFA-NRP1, but not SEMA3A-NRP1, signaling is required for angiogenesis (Gu et al., 2003). SEMA3E, a PLXND1 ligand, acts as a key repellent in endothelial cells (Fig. 6B), whereas VEGFA is a major attractant (Gu et al., 2005; Adams and Eichmann, 2010). RHOJ, a CDC42 subfamily GTPase, acts downstream of PLXND1 and is required for SEMA3E-induced endothelial cell repulsion. Although SEMA3E-PLXND1 signaling does not require NRP1 in angiogenesis, RHOJ coordinates interactions among PLXND1, NRP1, and VEGFR2, and controls their endocytic trafficking and degradation (see Fig. 4B) (Fukushima et al., 2020). Thus, RHOJ integrates VEGFA-mediated attractive and SEMA3E-mediated repulsive signaling.

Fluid shear stress is a major regulator in the cardiovascular system. In endothelial cells, PLXND1-mediated mechanotransduction contributes to the pathogenesis of atherosclerosis (Mehta et al., 2020). PLXND1 senses force by forming a mechano-sensing complex with NRP1 and VEGFR2 (Fig. 6B). In response to shear force, this complex promotes integrin signaling and focal adhesion formation. On the other hand, SEMA3s suppress integrin activation and focal adhesion formation, via neuropilins/plexins (Serini et al., 2003). Thus, PLXND1 acts as either a SEMA3E receptor or mechanosensor. SEMA3E-PLXND1 binding may prevent PLXND1 from entering the mechano-sensing mode, thereby suppressing pro-atherogenic responses of endothelial cells to mechanical stress (Mehta et al., 2020).

EFNB2–EPHB4-mediated repulsive, reciprocal signaling segregates arterial and venous precursor cells, organizing vessel networks (Wang et al., 1998). EFNB2 promotes VEGFR endocytosis, and the intracellular signals derived from VEGFA/VEGFR-carrying endosomes are essential for VEGFA-mediated filopodial extension of tip cells (Wang et al., 2010). Mechanistically, the endocytic sorting adaptor DAB2 and the cell-polarity regulator PAR3 interact with EFNB2 and VEGFR2/3, promoting VEGFR endocytic signaling (Fig. 4F) (Sigismund et al., 2021). Collectively, neuronal guidance genes have diverse activities in the cardiovascular system.

### Other organs

The important roles of neuronal guidance genes in organogenesis and homeostasis are also exemplified by studies of the muscle, the bone and the lung. Upon muscle development, mature myofibers are patterned by connecting with tendons. Myofibers undergo nerve innervation to establish the neuromuscular junctions (NMJs). Muscle-tendon attachment is essential for locomotion. SLIT, especially SLIT-N, is tethered onto tendon cells, acting as a repellent to direct muscle cell migration and elongation (Ordan et al., 2015). Guidance cues, especially netrins and semaphorins, regulate interactions between the presynaptic axon and target muscle at the NMJ (Carrillo et al., 2010). Muscle-derived SLIT2 acts as a retrograde signal that promotes presynaptic differentiation at the NMJ (Wu et al., 2015).

Bone homeostasis is maintained by the balance between bone-forming osteroblasts and bone-resorbing osteoclasts. Imbalances between bone formation and resorption lead to bone disorders, including osteoporosis. SEMA4D on osteoclasts suppresses osteoblast motility and bone formation (Negishi-Koga et al., 2011). By contrast, SEMA3A, secreted by osteoblasts, is an anti-osteoclastic ligand (Hayashi et al., 2012). SEMA3A also stimulates osteoblast differentiation in autocrine/paracrine manners, thereby promoting bone formation. Consistently, *Sema3A* knockouts exhibit osteopenic phenotypes. In addition, SEMA3A administration promoted bone regeneration in animal models (Hayashi et al., 2019). Therefore, SEMA3A is a candidate therapeutic target for bone diseases.

*SLIT2* and *SLIT3* are highly expressed in the lung (Wu et al., 2001). Attractive SLIT-ROBO signaling induces clustering of pulmonary neuroendocrine cells (PNECs) at branch points (Branchfield et al., 2016). The PNEC clusters maintain the branched structure of the lung and control inflammatory responses of immune cells, including macrophages. In *Robo1/Robo2* double knockout mice, the lung showed simplified alveolar structure (Branchfield et al., 2016), suggesting the involvement of SLIT-ROBO signaling in alveolar formation. Thus, ongoing research has revealed that neuronal guidance genes play versatile roles in the morphogenesis of muscle, bone, and other organs by regulating cell migration and cell-cell communications.

## Neuronal guidance genes in diseases and therapeutic strategies

Dysregulation of neuronal guidance genes has been implicated in various developmental, neuropsychiatric, and neurodegenerative disorders (Van Battum et al., 2015). The pathogenic mechanisms by which mutations in, or dysfunction of, neuronal guidance genes cause human diseases are largely unclear. Here, we discuss how defects in neuronal guidance contribute to pathogenesis and review recent advances in therapeutic approaches.

### Diseases associated with NTN pathways

Several human diseases have been associated with defective netrin signaling. Disorder of congenital mirror movements (CMM) is characterized by involuntary movements on one side of the body that mirror voluntary movements on the opposite side. Individuals with CMM are often carriers of a heterozygous pathogenic variant in *DCC*, *NTN1*, or the DNA recombinase *RAD51* (Srour et al., 2010; Depienne et al., 2012). Abnormalities of the corpus callosum have been detected in individuals carrying such a heterozygous variant in *DCC*. Biallelic loss-of-function *DCC* mutations were found in individuals with split-brain syndrome and associated with disorganization of white-matter axon tracts, including the complete loss of commissural axon tracts (Jamuar et al., 2017). Therefore, DCC play critical roles in organizing the white-matter architecture, including commissural formation. In addition, genetic variations or disruption in *NTNG1*/*2* have been associated with schizophrenia, bipolar disorder, and Rett syndrome (Woo et al., 2009).

Since its identification, DCC has been a candidate tumor suppressor. As a dependence receptor, DCC induces apoptosis in the absence of netrin-1, while netrin-1-DCC binding suppresses apoptosis (Mehlen et al., 1998). However, precise roles of DCC in cancer remain controversial.

A role of astrocytic NEO1 in protecting against epilepsy has been reported recently. Astrocyte-specific *Neo1* deficiency led to increased seizure in mice; inhibitory synaptic vesicles and GABAergic synaptic transmission were reduced in the hippocampus. Consistently, *NEO1* expression was decreased in hippocampal samples of epilepsy patients (Sun et al., 2021). However, roles of netrins in this NEO1-mediated process remain to be understood.

Bearing a death domain (Fig. 1), UNC5s may also act as dependence receptors. Apoptotic netrin-UNC5 signaling has been linked to neurodegenerative diseases, including Alzheimer’s disease (AD) and Parkinson’s disease (PD). Netrin-1 is neuroprotective for SN dopamine neurons and its administration *in vivo* restores their axon projections (Jasmin et al., 2021). Mutations/variations in UNC5C have been associated with late-onset AD (Wetzel-Smith et al., 2014). Cleavage of UNC5C by δ-secretase increases proapoptotic UNC5C activity, promoting AD pathoogy (Chen et al., 2021). Thus, novel strategies modulating netrin signaling may be developed for AD and PD.

### Diseases associated with SLIT pathways

The SLIT-ROBO pathway has been implicated in neuropshychiatric diseases. *De novo* mutations in *SLIT2*/*3* have been identified in schizophrenia patients (Gulsuner et al., 2013). Sex difference in depression susceptibility has been recognized, but the underlying mechanisms remain unclear. A recent study showed female-specific downregulation of *SLIT1* mRNA in the ventromedial prefrontal cortex (vmPFC) of patients with major depressive disorder. *Slit1* knockdown decreased dendritic arborization of vmPFC neurons and neuronal excitability in female, but not male, mice. RNA-seq analyses detected stress signatures in the *Slit1*-knockdown female mice (van der Zee et al., 2022). *ROBO3* mutations accompanying defects in axon midline crossing in the hindbrain have been detected in patients with horizontal gaze palsy with progressive scoliosis (HGPPS) (Jen et al., 2004).

Congenital diaphragmatic hernia (CDH) is a common disease. *Slit3*-deficient mice exhibit defects in diaphragm development, reminiscent of CDH (Yuan et al., 2003). *Robo1*/*2* double knockout mice exhibit delays of foregut tube separation from the body wall, leading to foregut mispositioning and diaphragm defects (Domyan et al., 2013). Consistently, genomic lesions encompassing *ROBO* genes are found in CDH patients. Therefore, SLIT-ROBO signaling regulates morphogenesis of the foregut and the diaphragm.

Loss-of-function *ROBO1* variants have been linked to Tetralogy of Fallot, a congenital heart disease. Mouse models carrying *Robo1* missense or null mutation reproduced phenotypes of ventricular septal and outflow tract defects (Kruszka et al., 2017). Although roles of *ROBO4* in developmental angiogenesis remains controversial, *ROBO4* variants are linked to a congenital defect, the bicuspid aortic valve, which frequently accompanies thoracic aortic aneurysm (Gould et al., 2019). Collectively, dysregulation of SLIT-ROBO signaling contributes to various developmental and neuropsychiatric disorders.

### Diseases associated with SEMA and EFN pathways

Semaphorins and their receptors have been implicated in neurological, cardiovascular, and inflammatory diseases (Suzuki et al., 2008; Worzfeld and Offermanns, 2014). Heteroinsufficiency of *SEMA3A* or *PLXNA1* has been implicated in Kallmann syndrome, a genetic disorder with delayed or absent puberty and hypogonadism, accompanied by decreased odor detection (Van Battum et al., 2015). Semaphorin pathways regulate the balance between excitatory and inhibitory synaptic transmission. Their imbalance has been implicated in neurological diseases, including epilepsy, learning disability, and cognitive disorders. Semaphorin-mediated homeostatic scaling is compromised in these diseases (Van Battum et al., 2015).

Semaphorin and ephrin signaling pathways utilize the chimaerin subfamily of RAC1-GAPs. Mutations in two chimaerin genes, *αChn* (*CHN1*) and *βChn* (*CHN*2), have been implicated in several diseases. CHN1 mediates ephrin-triggered corticospinal axon repulsion, thereby preventing midline recrossing of these axons (Iwasato et al., 2007). A spontaneous mutation in *Chn1* causes a hopping gait phenotype in mice. In humans, hyperactive *CHN1* variants have been linked to a congenital eye movement disorder, Duane’s retraction syndrome (Miyake et al., 2008). SEMA3F-triggered, CHN2-mediated RAC1 inactivation is required for hippocampal infrapyramidal tract pruning and synapse elimination (Riccomagno et al., 2012).

Several developmental and neurodegenerative diseases have been associated with ephrin-EPH pathways. For example, mutations in *EFNB1* were identified in craniofrontonasal syndrome (CFNS). Dysregulation of tissue boundary formation at the coronal suture has been proposed for CFNS pathogenesis (Twigg et al., 2004). *EPHA1* has been identified as a risk gene for late-onset AD (Karch and Goate, 2015). *EPHA4* has been proposed as a disease modifier gene of amyotrophic lateral sclerosis (ALS). In ALS patients, *EPHA4* expression levels correlate inversely with disease onset and survival, whereas loss-of-function *EPHA4* variants are linked to longer survival (Van Hoecke et al., 2012).

Increasing evidence supports the roles of semaphorins in modulating immune and inflammatory responses in multiple organs (Suzuki et al., 2008; Kanth et al., 2021). Among them, SEMA7A, presented on T cells, activates macrophages and drives the effector phase of inflammation (Suzuki et al., 2008). Interestingly, SEMA7A also promotes the resolution of acute severe inflammation, such as sepsis, by stimulating macrophage clearance and thus supporting tissue repair (Körner et al., 2021). On the other hand, SEMA7A also activates platelets and induces thrombo-inflammation caused by reperfusion injury after myocardial ischemia (Köhler et al., 2020). The formation of the platelet-neutrophil complex has been implicated in thrombo-inflammation processes. Pharmacological modulation of SEMA7A activity may have therapeutic potentials for diseases associated with inflammation.

### TENs and LPHNs in neuropsychiatric disorders

Mutations and single-nucleotide polymorphisms in *Teneurin 4* and *Latrophilin* genes have been associated with bipolar disorder and schizophrenia (Croarkin et al., 2017; Südhof, 2017; Yi et al., 2021). *LPHN3* variants have been implicated in attention deficit hyperactivity disorder (ADHD) (Franke et al., 2012).

### Tumorigenesis and cancer metastasis

Neuronal guidance genes may suppress or promote tumorigenesis, cancer progression or metastasis by affecting cell–cell communications within tumors or between tumor cells and microenvironment (Mehlen et al., 2011; Worzfeld and Offermanns, 2014; Toledano et al., 2019; Janes et al., 2021). There is extensive literature covering involvements of canonical and non-canonical guidance genes in cancer. Here, we only discuss a few examples in SLIT and semaphorin pathways to show complex roles of neuronal guidance genes in tumorigenesis and cancer metastasis.

Expression of *SLIT2* or *ROBO1* is frequently decreased in different types of cancer, due to promoter hypermethylation or loss of heterozygosity (Dallol et al., 2002; Tseng et al., 2010). Reduced expression of *SLIT2* is associated with shorter survival of lung cancer patients (Kong et al., 2015; Tavora et al., 2020). SLIT2-ROBO1 signaling inhibits RhoGAP activity of MYO9B and increases RHOA activity (Fig. 6C), which suppresses lung tumor growth and metastasis in mice (Kong et al., 2015). On the other hand, metastatic tumors may induce differential expression of *SLIT2*, with higher expression in endothelial cells relative to tumor cells (Tavora et al., 2020). Mechanistically, endogenous retroviral element-derived double-stranded RNAs are released by metastatic cancer cells, activate an innate-immune mechanism and upregulate *SLIT2* in endothelial cells. SLIT2, secreted by endothelial cells, acts as a chemoattractant for ROBO1-expressing cancer cells. Such SLIT-mediated communications between cancer and endothelial or stromal cells may regulate metastasis, because endothelial *Slit2* deficiency suppresses metastasis, whereas *Slit2* deficiency in tumor cells promotes metastasis (Fig. 6D) (Tavora et al., 2020).

In addition, in mice, SLIT-ROBO signaling inhibits tumor growth by suppressing the CXCL12-CXCR4 axis (Marlow et al., 2008 and references within). Loss of *Slit2*/*3* upregulates *Cxcl12* in mammary epithelium and the surrounding stroma, as well as *Cxcr4* in the mammary epithelium, suggesting inhibitory roles of SLITs in cancer progression.

Importantly, *SLITs* or *ROBOs* are mutated in ~15% of pancreatic cancer patients (Biankin et al., 2012). Reduced *ROBO2* expression and increased *ROBO3* expression is associated with poor survival in patients with pancreatic ductal adenocarcinoma (PDAC). Furthermore, ROBO2 acts as a stromal suppressor of PDAC by repressing myofibroblast activation and T-cell infiltration (Pinho et al., 2018). These data suggest that dysregulated SLIT signaling contributes to pancreatic cancer progression.

Tumor microenvironment (TME) is critical for cancer progression and metastasis. TME is maintained by crosstalk among neoplastic, stromal, endothelial and immune cells. Chronic inflammation is a hallmark for cancer. It has become clear that neuronal guidance genes modulate behaviors of not only tumor cells but also other cells in TME. For example, several SEMA3s (e.g. SEMA3A and SEMA3B) act as inhibitors of tumor progression, whereas SEMA3C exhibits both anti- and pro- tumorigenic effects. SEMA3A and SEMA3B not only suppress tumor angiogenesis but also regulate immune cells in TME, including tumor-associated macrophages (TAMs). SEMA3C also shows anti-angiogenic activity. However, expression of SEMA3C seems to correlate with tumor progression (Toledano et al., 2019). Furthermore, SEMA3C regulates the adhesion-detachment balance of tumor cells. Reduced SEMA3C signaling within tumors induces metastatic neuroblastoma dissemination (Delloye-Bourgeois et al., 2017). A deeper understanding of semaphorin signaling in TME may help in developing new therapeutic strategies against metastasis.

### Therapeutic potentials of modulating neuronal guidance signaling in neural repair

Neural regeneration and repair have been an area of active research for decades. However, effective treatments remain to be developed for either acute or chronic neural injuries. Reconstruction of neural networks by transplantation of stem cell-derived neurons or enhancement of adult neurogenesis may provide therapeutic potentials for neural repair (Barker et al., 2018). Although neural stem cells have been documented in adult mammalian brains, it remains controversial whether adult neurogenesis occurs in humans (Denoth-Lippuner and Jessberger, 2021). Several strategies, possibly in combination, will be necessary for effective neural repair, including rescuing injured axons from further damage, reducing neuronal death, guiding the migration of transplanted or newly generated neurons to appropriate target sites and ensuring proper axon rewiring of surviving neurons and adult-born neurons (Winter et al., 2021). One problem is that the axon growth-inhibitory environment in the adult brain limits regeneration after damage or disease. Another issue is that secondary events following the initial injuries, including oxidative stress and inflammation, cause further damage. Although modulating neuronal guidance genes may be useful for restoring neural circuits, most approaches have only been examined using animal models. Below we discuss several research trials for neural repair.

A major barrier that prevents neural recovery is exracellular inhibitors of axon regrowth produced by oligodendrocytes, such as NOGO-A (RTN4; NOGO-66 corresponds to its extracellular active region) (Fig. 1) and myelin-associated glycoprotein (MAG). These are ligands for RTN4R (NGR1). In a pre-clinical study, RTN4R decoy fused to Fc fragments of IgG1, intrathecally administered in primate models of spinal cord injury, promoted corticospinal axon regrowth (Wang et al., 2020). At the site of spinal cord injury, elevated levels of WNTs create non-permissive environments. Suppression of repulsive WNT-RYK signaling facilitated functional recovery from spinal cord injury and remapping of the motor cortex (Hollis et al., 2016). Anti-RYK antibody infusion also improved recovery from spinal cord injury in rodent models. Furthermore, WNT-directed manipulation coupled with rehabilitation promoted adult neurogenesis and trophic factor production, showing potential for treating spinal cord injury (Zou, 2020b).

Therapeutic strategies incorporating transplantation of neural stem cell-derived progenitors are being developed for neural injury treatments. However, neural progenitor migration is hampered by reactive glia. Upon transplantion, SLIT-expressing progenitors disrupted contacts between themselves and reactive astrocytes, allowing neurons to migrate to injury sites and promoting functional recovery of the post-stroke mouse brain (Kaneko et al., 2018). Interestingly, another recent study revealed that increased *Slit2* expression protected adult neurons against ischemia in a transgenic mouse model. SLIT2 enhanced glymphatic clearance, reduced peri-infarct neuroinflammation, increased the number of GABAergic interneurons, and improved cognitive function (He et al., 2020). Consistent with a potentially neuroprotective role of SLIT-ROBO signaling, SLITs, together with ROBO1/2, are highly expressed in subpopulations of adult human brain neurons, as shown by single-cell RNA-seq analyses (unpublished observation). Combinatorial approaches of cellular manipulation of SLIT-ROBO signaling at neuron-glia interfaces may hold promise for neural repair.

Furthermore, pharmacological manipulation of semaphorin signaling in homeostatic scaling and neural remodeling may provide new therapeutic applications for epilepsy and brain damage. Encouragingly, one drug is entering phase II clinical trials. In primate models, orally administered edonerpic maleate, a chemical compound that binds to CRMP2, a signal transducer of semaphorin pathways (Goshima et al., 1995), promotes functional recovery from brain damage; and edonerpic maleate upregulates AMPAR trafficking to synapses (Abe et al., 2018). These approaches targeting neuronal guidance, including modulating neuron-glia communications, neural rewiring, and homeostatic scaling, may provide novel therapeutic strategies for neurodevelopmental/neuropsychiatric diseases and neural injuries.

## Conclusion and perspective

Remarkable progress has been made in molecular and functional characterization of neuronal guidance genes and signaling pathways that regulate cue sensing, receptor dynamics and cytosketal reorganization during axon pathfinding and cell migration. A few fundamental concepts have emerged. First, expression and function of neuronal guidance cues, receptors and downstream signal transducers are under spatiotemporal control at multiple levels. Second, guidance genes regulate cellular responses in various ways. The same genes can act in multiple places/times with different functions (e.g. NTNs, SLITs, and SEMAs). The same proteins can act as both ligands and receptors (e.g. EFNs, EPHs, TENs, and LPHNs). The ligands can act as secreted or immobilized molecules to attract or repel axons or neurons in context-dependent manners. Guidance cues can be presented by other cells not producing these cues. Third, neuronal guidance signaling also utilizes the cell-adhesion machinery. Fourth, the guidance system can regulate both short- and long-range cell–cell communications. Fifth, there are extensive cross-communications among different guidance cues, receptors, and downstream signal transducers. Sixth, neuronal guidance genes play important roles in synaptogenesis, plasticity, and circuit formation. Furthermore, neuronal guidance signaling acts together with neural activity in the formation and refinement of the complex neural architecture. Finally, neuronal guidance genes regulate cell migration and cell–cell communications outside the nervous system, thereby regulating organogenesis and tissue homeostasis. Aberrant guidance signaling in various cell types and tissues has been implicated in a wide range of human diseases, from developmental, neuropsychiatric, and neurodegenerative disorders to cancer metastasis.

Despite such advances, significant gaps remain in our knowledge regarding the physiological roles of neuronal guidance genes. It is still unclear how the establishment of the immensely complex nervous system is regulated by relatively small classes of neuronal guidance genes. We have just begun to catch a glimpse of the molecular principles by which multiple signaling pathways of neuronal guidance exert both local and long-range effects on cell behaviors, while enabling cells to adapt to the constantly changing environment. We know little about mechanistic bases by which individual cells integrate signals from a myriad of different guidance ligands, cell-adhesion molecules, and other environmental cues. Even less is known about principles of information processing and integration at the neural circuit, organ, and organismal levels. Importantly, much of our knowledge about neuronal guidance is from studying animal models. We only have limited tools to study functional roles of neuronal guidance genes in human pathophysiology.

Emerging RNA-sequencing data show that many neuronal guidance genes are highly expressed in distinct populations of adult human neurons and non-neuronal cells, suggesting potential roles of these genes in maintenance and/or function of the human brain. Furthermore, recent efforts to construct a brain cell atlas and census, in which higher-resolution single-cell spatial transcriptomic imaging is combined with multimodal analyses for morphological and electrophysiological annotations, should provide us with a “treasure trove” of detailed and integrated information (BICCN, 2021). With combined applications of genetics, genomics, transcriptomics, and proteomics tools, together with new imaging modalities, functional, and behavioral assays in comparative analyses of animal models and human samples, novel functions and signaling mechanisms of neuronal guidance genes will be uncovered. Such multi-pronged approaches will provide information valuable for future development of therapeutic approaches for treatments of neural injuries and diseases associated with mutations in, or dysfunction of, neuronal guidance genes.

## Abbreviations

AD: Alzheimer’s disease
ADHD: attention deficit hyperactivity disorder
ALS: amyotrophic lateral sclerosis
AMPAR: AMPA-type glutamate receptor
BAI: brain-specific angiogenesis inhibitor
CA: cornu ammonis
CAM: cell-adhesion molecule
CDH: congenital diaphragmatic hernia
CFNS: craniofrontonasal syndrome
CHD: congenital heart disease
CMM: congenital mirror movements
DA: dopamine
DG: dentate gyrus
DV: dorsal-ventral
ECD: extracellular domain
FLRT: fibronectin leucine-rich repeat transmembrane protein
FP: floorplate
GAP: GTPase-activating protein
GEF: guanine nucleotide exchange factor
GPCR: G protein-coupled receptor
GPI: glycosylphosphatidylinositol
HSC: hematopoietic stem cell
ICD: intracellular domain
Ig: immunoglobulin
LCR: low-complexity region
MAG: myelin-associated glycoprotein
MAPK: mitogen-activated protein kinase
NMJ: neuromuscular junction
NTN: netrin
OB: olfactory bulb
OE: olfactory epithelium
OR: odorant receptor
OSN: olfactory sensory neuron
PCP: planar cell polarity
PD: Parkinson’s disease
RC: rostral-caudal
RGC: retinal ganglion cell
RTK: receptor tyrosine kinase
SAC: starburst amacrine cell
SC: superior colliculus
SHH: sonic hedgehog
SN: substantia nigra
SNc: substantia nigra pars compacta
SNr: substantia nigra pars reticulata
TNT: tunnelling nanotube
TZ: termination zone
VEGFA: vascular endothelial growth factor A
VSD: ventricular septal defect
VZ: ventricular zone

## Declarations

J.Y.-K., M.K.-K. and Y.T. have been supported by JST, JSPS and AMED; J.Y.W. has been supported by the NIH. All the authors of this paper declare that they have no conflict of interest. Animal experiments were carried out according to protocols approved by the Institutional Animal Care and Use Committees (IACUC) of Northwestern University. All data that support the findings of this study are included in the manuscript. All other data and materials are available from the corresponding authors upon reasonable request.

All the authors participated in the design of manuscript conception and outline, and contributed to manuscript editing.
